# Impact of Thermophysical and Biological Pretreatments on Antioxidant Properties and Phenolic Profile of Broccoli Stem Products

**DOI:** 10.3390/foods13223585

**Published:** 2024-11-10

**Authors:** Claudia Bas-Bellver, Cristina Barrera, Lucía Seguí

**Affiliations:** Institute of Food Engineering—FoodUPV, Universitat Politècnica de València, Camino de Vera, s/n, 46022 Valencia, Spain; clbabel@upv.es (C.B.-B.); mcbarpu@tal.upv.es (C.B.)

**Keywords:** broccoli stem, waste valorisation, dehydrated powders, pretreatments, fermentation, ultrasounds, microwaves, antioxidants, functional ingredient

## Abstract

Fruit and vegetable industrialisation is a major contributor to food waste; thus, its integral transformation into functional powders has gained attention. Pretreatments can be incorporated into valorisation processes to generate structural or biochemical changes that improve powders’ characteristics. This study deepens into the impact of biological (fermentation, FERM) and thermophysical (autoclaving, AUTO; microwaves, MW; ultrasound, US; and pasteurisation, PAST) pretreatments, combined with dehydration (hot air-drying, HAD; or freeze-drying, FD) on the characteristics of powdered products obtained from broccoli stems. The impact of pretreatments on physicochemical (moisture, water activity, total soluble solids) and antioxidant properties (phenols, flavonoids, antioxidant capacity by ABTS and DPPH) on residue and powdered products was studied, together with their impact on plant tissue structure (Cryo-SEM) and the powders’ phenolic profile (HPLC). Probiotic viability was also determined on the fermented samples. The pretreatments applied, particularly the ultrasound, improved the antioxidant properties of the broccoli stems compared to the unpretreated samples, in line with microscopic observations. Dehydration did also improve the antioxidant attributes of the broccoli wastes, especially drying at 60 °C. However, pretreatments combined with dehydration did not generally lead to an improvement in the antioxidant properties of the powders. Probiotic properties were preserved in the freeze-dried products (>10^7^ CFU/g). In conclusion, pretreatments may be applied to enhance the antioxidant attributes of broccoli wastes, but not necessarily that of dried powdered products.

## 1. Introduction

The food industry generates a large amount of waste, a current issue related to the scarcity of natural resources [1], with waste being of major relevance in the horticulture industry [2]. It has been estimated that around 45% of total fruit and vegetable production is lost each year during the various stages of the food chain, from fields to processing and consumption [3].

For many years, the continuous production of waste has been encouraged by a linear economy design of food systems, increasing the discharge of waste to the environment, and ending its passage through the food chain. With the 2030 Agenda for Sustainable Development, it is proposed that the linear economy is to be replaced by a circular economy design, which comprises all activities related to the reintroduction of waste into production, distribution, and consumption processes [1], contributing to the achievement of the Sustainable Development Goals (SDGs).

One of the most widely grown cruciferous vegetables in the world is broccoli (*Brassica oleracea* var. *italica*) [4], with a global production higher than 26 million tonnes, together with cauliflower, in 2022 [5], which is directly related to a disproportionate increase in waste generation. Commonly, only the broccoli florets are used for food purposes, so in the fresh-cut or frozen industry, 47% of broccoli is classified as waste (stem and leaf) [4], despite its richness in minerals, vitamins, bioactive compounds (such as glucosinolates, phenolic compounds, and carotenoids), and antioxidant properties that can prevent oxidative stress that contributes to pathogenesis [6,7,8].

Fresh stems have a high moisture content, which promotes spoilage [3,9], for which broccoli residues need to be processed into products with improved stability and shelf life. One of the approaches to valorise these waste products is the production of powdered functional ingredients through processes based on disruption, dehydration, and milling [10]. Vegetable powders may have applications as colouring or flavouring agents, as well as in fortifying foods and improving their nutritional value [7,11,12,13].

Hot air-drying (HAD) is one of the most widely used dehydration techniques to increase the stability of foods and allow their proper preservation [14]. However, the characteristics of the resulting product depend on the process variables [15,16]. Another method used in product preservation is freeze-drying (FD), which is gaining relevance in food stabilisation as it leads to high-quality products due to the low temperature and vacuum conditions applied [17]. The response of vegetal material to drying can be modified by the application of pretreatments, which may improve water diffusion through the tissue and reduce drying times [18]. Additionally, pretreatments cause tissue disruption, which facilitates the extraction of bioactive compounds and the release of enzymes that promote biochemical reactions, generating more active forms and thereby increasing the bioactivity of certain compounds [19]. Pretreatments can also contribute to the release of active compounds which are naturally bound to cellular structures [20]. One of the simplest pretreatments that can be applied to facilitate water transfer and release compounds of interest is physical tissue disruption. The impact of grinding intensity, as well as freeze–thaw pretreatment, have already been evaluated in previous studies [10,11]. In the present work, other pretreatments which have the potential to modify plant structures are investigated.

Biological treatments include enzymatic and microbial treatments. Vegetables’ fermentation with lactic acid bacteria is a common practice in the food industry. Fermentation may modify the vegetable matrix and release antioxidant compounds. Lactic acid bacteria (LAB) fermentation allows the transformation of polyphenols and other bioactive compounds into more bioactive forms through the production of certain enzymes [21,22,23]. Among LAB, *Lactiplantibacillus plantarum*, a QPS-status microorganism, has been proposed for broccoli fermentation due to its ability to grow in this plant matrix [23,24].

Thermophysical pretreatments include classical and emerging technologies. In this case, the structure of the plant tissue might be modified due to a high temperature or pressure, as in the case of autoclave or pasteurisation treatments, or due to the response of the interaction of the plant material with mechanical (ultrasounds) or electromagnetic waves (microwaves). The interaction with microwaves generates thermal and non-thermal effects which may cause structural changes due to the vapour explosion generated in overheating points (hotspots). The interaction of the plant matrix with electromagnetic waves accelerates physicochemical reactions by heating, which, together with structural modifications, makes it possible to reduce the drying time, improving the nutritional profile of the products [25,26]. Ultrasound is an emerging technology increasingly applied in food product and process research which, combined with drying, can reduce energy expenditure and result in final products with improved properties [27,28]. It is known that ultrasound pretreatment shortens the drying time for both HAD and FD [29] due to the so-called ‘sponge effect’, which enhances the intracellular water transfer to the surface [27], as well as the dispersion of intracellular components [30]. Pasteurisation and autoclaving are thermal pretreatments widely used in the food processing industry [31]. On the one hand, autoclaving is performed under high pressure and temperature conditions, which have been linked to cell wall breakage due to cellulose solubilisation [32]. Thus, damage to cell walls can promote the extraction of bioactive compounds and increase their bioaccessibility [31]. Pasteurisation has been proposed for the inactivation of undesired enzymes, as well as to preserve the nutritional content of food products [33]; furthermore, the use of this pretreatment is also justified by the need of reducing microbial loads prior to fermentation.

The aim of this work was to evaluate the impact of different pretreatments (microwaves, ultrasounds, autoclave, pasteurisation, and fermentation with *L. plantarum*) and drying techniques (HAD and FD) on the physicochemical, antioxidant, and structural properties of broccoli ground stems, as well as to evaluate their convenience as pretreatments to obtain sustainable broccoli powdered ingredients with improved functional properties.

## 2. Materials and Methods

### 2.1. Raw Material

Broccoli (*Brassica oleracea* var. *italica*) heads were purchased from a local supermarket in Valencia (Valencia, Spain) and the stems, which corresponds with the IV range or frozen bags waste, were manually separated using a knife. The fresh broccoli stems were washed with a 1% (*v*/*v*) sodium hypochlorite in water solution. After that, the stems were disrupted in a food processor (Thermomix^®^, Vorwerk, Madrid, Spain) for 8 s at 10,000 rpm [34] before undergoing the processes described below.

### 2.2. Inoculum Preparation

*Lactiplantibacillus plantarum* spp. CECT 749 (Colección Española de Cultivos Tipo, Valencia, Spain) was used to inoculate the broccoli waste. This microorganism was selected on the basis of its potential probiotic effect [35], its ability to degrade different polysaccharides, and its metabolic diversity and adaptability [36].

The freeze-dried strain was reactivated in Man, Rogosa, and Sharpe (MRS) broth (Scharlab, Barcelona, Spain) and incubated at 37 °C for 24 h (Incugidit, PSelecta, Barcelona, Spain), as described in [34]. The starter culture obtained contained 9.4 ± 0.2 log CFU/mL (according to plate count measurements). All materials used for microbiological analyses were conveniently sterilised in an autoclave at 120 °C for 2 h (Systec GmbH VB-40, Linden, Germany).

### 2.3. Powder Manufacturing

#### 2.3.1. Preliminary Fermentation and Drying Study

**Figure 1:**
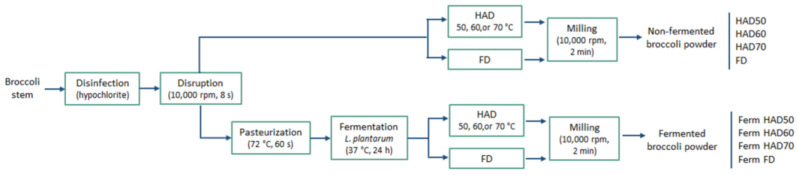
Process flow diagram for the integral transformation of broccoli stems into non-fermented and fermented powdered products. HAD: hot air-drying; FD: freeze-drying; Ferm: fermentation.

A preliminary study was carried out to evaluate the effect of fermentation and drying on the properties of powdered products. Powders were obtained by HAD at 50, 60, or 70 °C or FD, from fermented and unfermented ground broccoli stems (Figure 1). To reduce the variability due to the sample origin, all powders were obtained from the same batch. The broccoli stems were ground and mixed, distributed as explained next, and processed simultaneously.

To proceed with fermentation, ground broccoli residue was pasteurised in a hot water bath to reduce the initial microbial load. For this aim, the sample was distributed in glass jars containing 200 g of the ground residue, which were immersed in a hot water bath at 82 °C, until reaching a temperature of 72 °C in the geometric centre, and maintained for 1 min. A total of 2 mL of the prepared inoculum (~10^9^ CFU/mL) was added to each glass jar containing 200 g of broccoli and incubated at 37 °C for 24 h. All the material used in this process was previously sterilised in an autoclave (Systec GmbH VB-40, Linden, Germany) at 120 °C for 2 h.

HAD and FD were used as dehydration techniques. In this preliminary study, HAD was carried out on a laboratory scale, in three temperature-controlled bench tray dryers (Gastroback, Natural Plus 46600, Hollenstedt, Germany), each one at a corresponding temperature (50, 60, or 70 °C). The sample was prepared in 1 cm-thick layers and placed on the drier-perforated trays with a load of 200 g/tray. HAD was carried out with a constant air temperature of 50, 60, or 70 °C until a a_w_ value below 0.3 was reached, to guarantee the stability of the dehydrated residue [37]. For FD, fermented and unfermented residues were distributed in 1 cm-thick layers in aluminium trays. The samples were first deep-frozen at −40 °C in a CVN-40/105 freezer (Matek, Barcelona, Spain) and next freeze-dried for 48 h (LyoQuest-55 freeze-dryer, Terrasa, Spain) at −45 °C (condenser temperature) and at a sub-atmospheric pressure (0.1 mbar). After dehydration, either by HAD or FD, the dried residue was milled in a Thermomix^®^ food processor (10,000 rpm for 2 min in 30 s intervals) [34] to yield a fine powder. The powders were then stored in twist-off glass jars in a light-free environment at room temperature until analysis.

#### 2.3.2. Impact of Thermophysical and Biological Pretreatments on Ground Broccoli Stems and Powdered Products

**Figure 2:**
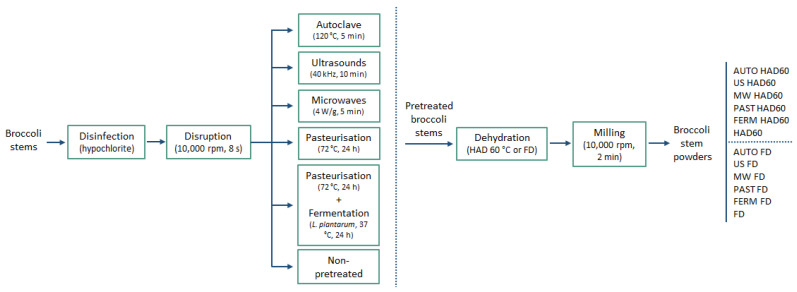
Process flow diagram for the transformation of broccoli stems into pretreated wastes and corresponding dried powders. HAD: hot air-drying; FD: freeze-drying; Ferm: fermentation.

Thermophysical and biological pretreatments were applied to the residue prior to dehydration (Figure 2). Again, all the raw material used in this series of experiments came from one batch, and was ground and processed simultaneously, to reduce variability due to the sample origin. Thus, the broccoli ground waste was distributed into sterile twist-off glass jars (200 g per jar) and subjected to the corresponding pretreatment, which were the following: autoclave at 120 °C for 5 min (Systec GmbH VB-40, Linden, Germany), microwave oven at 4 W/g for 5 min (Samsung GW72N, Samsung Electronics, Suwon, Republic of Korea), ultrasound at 40 kHz for 10 min (Ultrasons-H, Selecta, Barcelona, Spain), and fermentation with *Lactiplantibacillus plantarum* for 24 h at 37 °C (Incugidit, PSelecta, Barcelona, Spain) after pasteurisation. In addition, fresh and pasteurised residues were used as controls. The selection of pretreatment conditions was based on the available literature [20], along with unpublished results from our own laboratory. Pasteurisation and fermentation processes were carried out as previously described (Section 2.3.1).

Fresh and pretreated samples were then dehydrated by HAD or FD. In this case, all HAD samples were dried under the same conditions and simultaneously to ensure the homogeneity of the treatment. Therefore, the HAD of the fresh and pretreated samples was carried out in a pilot plant convective transverse flow tray dryer (CLW 750 TOP+, Pol-Eko-Aparatura SPJ, Katowice, Poland) with air at 60 °C and an air velocity of 2 m/s for 10 h. The drying time and temperature were decided based on the results of preliminary test. Samples were spread in 1 cm-thick layers on perforated dryer trays with a load of 200 g/tray (2 trays/pretreatment). The FD, milling, and storage of powders were performed as explained earlier (Section 2.3.1).

### 2.4. Characterisation of Intermediate and Final Broccoli Stem Products

#### 2.4.1. Microbial Counts

The viability of *Lactiplantibacillus plantarum* cells was determined by serial dilution (10^−1^ to 10^−8^) with sterile buffered peptone water (Scharlab, Barcelona, Spain) and subsequent surface seeding on MRS agar (Scharlab, Barcelona, Spain), followed by incubation at 37 °C for 24–48 h. The first dilution (10^−1^) was obtained by homogenising in stomacher equipment (Interscience, BagMixer^®^ 400 model, St Nom, France) for 2 min 3 g of solid sample (fresh residue or dehydrated powder) with 27 mL of sterile peptone water. After incubation, colonies present on the plates were counted.

#### 2.4.2. Physicochemical Properties: Water Activity, Moisture Content, and Soluble Solids

Water activity (a_w_) was obtained with an Aqualab^®^ 4TE dew point hygrometer at 25 °C (Decagon Devices Inc., Pullman, Washington, DC, USA). Moisture content (x_w_) was obtained as described in the official method of the AOAC 934.06 [38], based on the weight loss before and after drying in a vacuum oven (Vaciotem-T, JP Selecta, Barcelona, Spain) at 60 °C and 200 mbar to a constant weight. The mass fraction of total soluble solids (x_ss_) was calculated from the moisture content and the measurement of the Brix degrees read at 20 °C in a thermostatic Abbe refractometer NAR-3T (Atago, Tokyo, Japan). In the powders, measurements were obtained from an aqueous extract (1:10 (*w*/*v*) ratio).

#### 2.4.3. Antioxidant Properties: Total Phenols and Flavonoids and Antiradical Activity

Antioxidant compounds were extracted by mixing 4 g of undried sample or 0.5 g of powdered product with 10 mL of 80% (*v*/*v*) methanol/water solution, followed by shaking (WY-100 horizontal shaker, COMECTA, Barcelona, Spain) for 1 h and further centrifugation for 5 min at 10,000 rpm in an Eppendorf centrifuge (5804/5804R, Eppendorf SE, Hamburg, Germany).

Total phenols were measured by the Folin–Ciocalteu method [39,40]. Thus, 0.125 mL of the extract was mixed with 0.5 mL of bidistilled water and 0.125 mL of the Folin–Ciocalteu reagent (Scharlab S.L., Barcelona, Spain) and let react in darkness for 6 min. Then, 1.25 mL of a 7% (*w*/*v*) sodium carbonate solution was added together with 1 mL of bidistilled water. Absorbance was measured at 760 nm, after 90 min in darkness, in a Cary 60 UV/Vis spectrophotometer. Results were given as mg of gallic acid equivalents (GAE) per g of dry matter, using gallic acid as the standard (purity ≥ 98%, Sigma-Aldrich, St Louis, MO, USA).

The total flavonoid content was determined following the modified aluminium chloride method [41]. A total of 1.5 mL of the extract and 1.5 mL of a 2% (*w*/*v*) aluminium chloride solution (Thermo Fisher Scientific Inc., Waltham, MA, USA) were mixed and reacted for 10 min in darkness. Absorbance was measured at 368 nm in a Cary 60 UV/Vis spectrophotometer. Results were given as mg of quercetin equivalents (QE) per g of dry matter, using quercetin as the standard (purity ≥ 95%, Sigma-Aldrich, St Louis, MO, USA).

The DPPH (1,1 diphenyl-2-picryl hydrazyl) and ABTS (2,20-azobis-3-ethyl benzothiazolin-6-sulphonic acid) methods were applied for determining the antioxidant activity of the samples. For the former [42], 0.1 mL of the extract was mixed with 2.9 mL of a 0.1 mM DPPH solution in methanol (Merck KGaA and affiliates, Darmstadt, Germany) and let react for 60 min in darkness. Then, absorbance was measured at 575 nm in a Cary 60 UV/Vis spectrophotometer (Agilent Technologies, Santa Clara, CA, USA). Results were expressed as mg of trolox equivalent (TE) per gram of dry matter. As for ABTS [43], 0.1 mL of the extract was mixed with 2.9 mL of an ABTS+ (VWR International LLC, Radnor, PA, USA) solution in phosphate buffer with an absorbance of 0.70 ± 0.02 at 734 nm. After 7 min of reacting, absorbance was measured at 734 nm in a Cary 60 UV/Vis spectrophotometer. Results were given as mg of trolox equivalent (TE) per g of dry matter, using trolox as standard (purity ≥ 97%, Sigma-Aldrich, St Louis, MO, USA).

#### 2.4.4. Phenolic Constituents by High Performance Liquid Chromatography (HPLC)

The phenolic profile of the powdered products was also determined. For this aim, the method proposed by Caprioli et al. [44] and Giusti et al. [45] was used, with some modifications. The methanolic extracts of the powders were prepared using an 80% (*v*/*v*) HPLC-grade methanol (Scharlab S.L., Barcelona, Spain) solution in double distilled water as a solvent at an extraction ratio of 1:10 (*w*/*v*). The mixture was shaken for 1 h in a horizontal shaker, kept in the dark, and centrifuged at 10,000 rpm for 5 min. The supernatants were collected and filtered through 0.45 µm PTFE filter (Scharlab S.L., Barcelona, Spain) and the resulting extract was analysed by HPLC. Two extracts per powder were prepared.

The equipment used for the HPLC analyses was HPLC 1200 Series Rapid Resolution equipment coupled to a diode detector (Agilent, Palo Alto, CA, USA). Separations were carried out on a Kinetex column (250 × 4.6 mm I.D., 5 μm; Phenomenex; Torrance, CA, USA) at 31 °C and using 1% formic acid (Scharlab S.L., Barcelona, Spain) as mobile phase A and acetonitrile (Scharlab S.L., Barcelona, Spain) as mobile phase B. The gradient used was as follows: 0 min, 90% A; 25 min, 40% A; 26 min, 20% A; held for 30 min; 35 min, 90% A, with holding for up to for 40 min. The injection volume was 10 µL and the flow rate was 0.5 mL/min. The phenolic compounds were identified by their retention times and spectra, as compared to HPLC reference standards (Extrasynthese, Genay, France), at a wavelength of 280 nm. The compounds were quantified by standard curves and results were given as mg/100 g_dm_.

### 2.5. Cryo-Scanning Electron Microscopy (Cryo-SEM)

The impact of the pretreatments on the microstructure of the ground broccoli stems was examined by cryo-SEM. For that purpose, a ZEISS ULTRA55 microscope (Carl Zeiss AG, Oberkochen, Germany) (Microscopy Service of the Universitat Politècnica de València), equipped with an external cryo-freezing chamber (OXFORD mod. CT-1500, Oxford, UK), was used. For the sample preparation, a small fragment of a fresh or fresh and pretreated ground broccoli stem was placed on the slide and fixed with a mixture of two components, colloidal graphite dispersion in water (G303 Colloidal Graphite AQUADAC, Agar Scientific, Stansted, UK) and a tissue fixative (Tissue-Tek AutoTEC^®^ a120, Sakura, Barcelona, Spain), to ensure proper fixation for subsequent fracture. The sample was placed in a cryo-chamber and frozen with liquid nitrogen (−210 °C) under vacuum conditions. The sample was fractured inside the observation chamber to expose the inner tissue microstructure. Subsequently, the sample was sublimated for 20 min at −85 °C with a pressure of 10^−5^ mmHg, after which a platinum coating was applied to provide the electron beam with a suitable reflecting surface. The coating or quorum sputtering was performed for 15 s. Finally, the samples were observed through the screen of a computer equipped with ZEISS SmartSEM software https://www.zeiss.com/microscopy/de/produkte/software/zeiss-smartsem.html (accessed on 12 September 2024) (Carl Zeiss AG, Oberkochen, Germany), at −150 °C and 10–20 kV. During tissue observation, micrographs were taken and saved at appropriate magnifications to allow sample comparison.

### 2.6. Statistical Analysis

Analytical determinations were performed in the powdered products obtained. Measurements were conducted at least in triplicate. Statistical analysis was carried out with Statgraphics Centurion XVIII software (version 17.1.04) (StatPoint Technologies, Inc., Warrenton, VA, USA). Analyses of variance (one-way ANOVA and multifactorial ANOVA) were performed with a confidence level of 95% (*p* < 0.05), after checking the data normality. A multiple range test using Fisher’s LSD method was performed to discriminate among the means and identify homogeneous groups.

## 3. Results and Discussion

### 3.1. Impact of Drying on Fermented and Non-Fermented Broccoli Stems

The results of the moisture content (x_w_), water activity (a_w_), and total soluble solids content (x_ss_) of the fresh, fermented, and dehydrated broccoli stems are shown in Table 1.

The moisture content and a_w_ values for the fresh and fermented samples were within the range obtained in previous studies [34] and implied a risk of spoilage, for which dehydration is justified. No significant differences were found between the fermented and non-fermented samples regarding moisture content and water activity. As observed, the soluble solids content remained constant after fermentation. This behaviour has also been observed in mixed mango and carrot juice fermented with *L. plantarum* [46], likely due to microorganisms’ action on more complex polysaccharides. During fermentation, bacteria degrade simple sugars but also use glycosidases and glycosyl hydrolases enzymes, among others, to breakdown polysaccharides from the plant matrix into sugar monomers that are more easily metabolised [46,47]. As expected, dehydration caused a significant reduction in the a_w_ and x_w_ (*p*-value < 0.05) to values which ensure a prolonged shelf life [48]. Fermentation caused a reduction in the drying times, since the processing time needed to reduce the a_w_ to safe values was as follows: 10 h at 50 °C, 7 h at 60 °C, and 5 h at 70 °C; this was in contrast to the drying of non-fermented broccoli residue which was completed in 12 h at 50 °C, 10 h at 60 °C, and 6 h at 70 °C. The fermented powders exhibited a higher moisture content than their non-fermented counterparts, a difference which could be explained by their structural breakdown and decompartmentalisation due to microbial action. However, this increase in moisture content was not observed in the water activity values, which were similar or slightly lower than in the non-fermented samples. These results suggest that microbial growth and metabolism modifies the way in which water interacts with the matrix, so fermented powders contain a higher proportion of bound water.

The soluble solids content of the powders was in the range of that reported in previous studies [10]. As evidenced in the literature, dehydration and subsequent milling may contribute to soluble solids’ increase due to the release of soluble compounds from broken cells and the breakage of fibres into simpler compounds [11,49,50]. This was observed in the FD powders, both fermented and non-fermented, which exhibited higher x_ss_ values (*p*-value < 0.05) than the HAD powders, a result which can be explained by the higher efficiency of milling in the FD samples due to the fragility and porous structure which is characteristic of FD products [14]. Thus, FD facilitates milling, resulting in a smaller particle size and promoting fibres’ breakdown and soluble compounds’ release. On the contrary, HAD can cause crusting phenomena, also known as case-hardening, which generates rubbery cores that make subsequent milling more difficult, resulting in powders with a lower soluble solids content [10,51,52].

**Figure 3:**
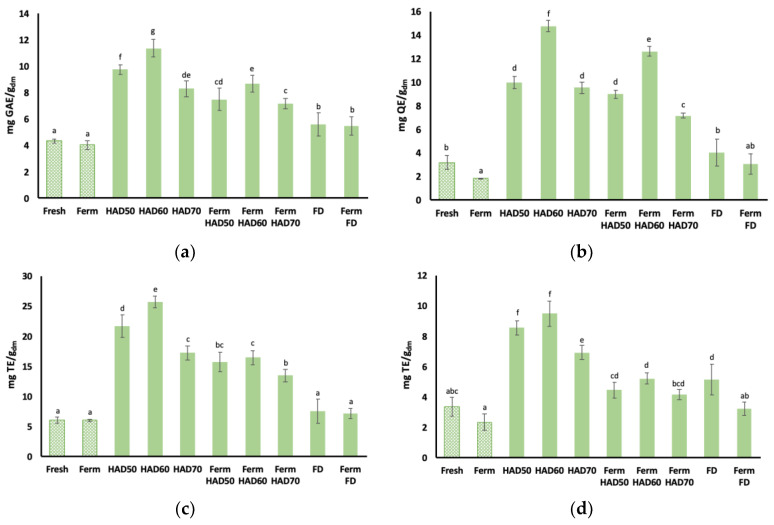
Antioxidant properties of fresh and fermented broccoli stems and their respective powders obtained by different dehydration techniques: (**a**) Total phenolic content expressed as mg gallic acid equivalent (GAE) per gram of dry matter. (**b**) Total flavonoid content expressed in mg quercetin equivalent (QE) per gram of dry matter. (**c**,**d**) ABTS and DPPH antioxidant capacity, respectively, expressed in mg trolox equivalent (TE) per gram of dry matter. HAD: hot air-drying at 50, 60, or 70 °C; FD: freeze-drying; Ferm: fermentation. Filled columns represent dehydrated samples, and dotted columns represent non-dried ones. Error bars correspond to standard deviation of six replicates from two replicas (3 replicates/replica). ^a–g^ Different superscript letters indicate statistically significant differences at 95% confidence level (*p*-value < 0.05), according to multiple range test.

Figure 3 shows the antioxidant properties values of the fresh and fermented stems before and after dehydration by the two techniques tested. Values are shown for the total phenol content (mg GAE/g_dm_), total flavonoid content (mg QE/g_dm_), and antioxidant capacity (mg TE/g_dm_) by the ABTS and DPPH radical methods. Among the non-dehydrated samples, only the total flavonoids content exhibited statistically significant differences, with a decrease in the fermented samples. Similar results were reported by Septembre-Malaterre et al. [47], who found a slight decrease in antioxidant properties (total phenol, flavonoid content, and DPPH scavenging activity) after the fermentation of cabbage with *L. plantarum* at 37 °C for 24 h. Similarly, Kiczorowski et al. [22] obtained a lower content of total phenols in fermented broccoli compared to an unfermented control. However, these results differ from those obtained in other studies. Li et al. [21] reported that *L. plantarum* improved the antioxidant capacity of apple juice, due to the consumption of glucose molecules available in phenolic compounds, thus generating metabolites with more hydroxyl groups or less steric hindrance. Also, Kwaw et al. [53] studied the impact of *L. plantarum* on the antioxidant activity of mulberry juice, finding that during fermentation there was a release of soluble phenolic compounds from the plant cell walls, increasing the antioxidant activity [54]. These disparities have already been discussed in the literature. According to Knez et al. [55], although one of the fundamental aspects of fermentation is the increase in antioxidant potential due to the release of phenols and other antioxidants from the plant matrix, the methodology for analysing antioxidant potential is not yet standardised, and there may be variations between the results of different studies. In addition, the fermentation of plant foods is a complex combination of factors in which interactions between microbiological, enzymatic, chemical, and biochemical reactions and physical processes occur [56], adding variability to determination methods and making it difficult to standardise procedures.

Antioxidant properties significantly increased for samples after the HAD and subsequent milling compared to their corresponding controls, but not for the FD samples. The multifactorial ANOVA considering the factors of fermentation and dehydration, and the interaction between them, revealed that both factors and their interaction were significant for the four antioxidant parameters analysed (*p*-value < 0.05) except for flavonoids, in which the interaction was not significant (*p*-value = 0.3450). For some drying conditions such as HAD there were statistically significant differences between the fermented and non-fermented samples, whereas these differences were not statistically significant when FD was applied, particularly for the total flavonoids and antioxidant capacities. The best results were obtained for the HAD60 powders, which exhibited higher values for all the antioxidant parameters analysed. However, increasing the temperature to 70 °C had a negative impact on antioxidant properties, as compared to lower temperatures. Similar results were reported by other authors in kiwifruit slices, in which the total phenol content increased significantly compared to a fresh control when drying at 60 °C [57]. The improvement of the antioxidant properties of vegetables after HAD has been reported by several authors [58,59,60,61]. Improved antioxidant properties can be explained by the formation of new antioxidant compounds as a result of biochemical reactions, such as Maillard reactions, due to exposure to high temperature [56,62] or isomerisations to more active forms [63], together with the reduction in certain pro-oxidant enzymes capable of degrading antioxidant compounds [15,16,51].

On the other hand, the Ferm HAD powders showed a decrease in antioxidant parameters, as compared to the dried samples which had not been fermented prior to dehydration (HAD). Differences between the Ferm HAD and HAD powders were more significant than differences between the fermented and non-fermented fresh residue. One possible explanation could be related to reducing sugars’ consumption by microorganisms [64], which could decrease Maillard reactions’ incidence. The decreased antioxidant properties of the Ferm HAD powders could also be attributed to the action of bacterial enzymes, which would have released antioxidant compounds from the structures [65], which would then become more exposed to oxygen and high temperatures during HAD. Differences between the fermented and non-fermented samples after HAD were statistically significant in all cases, but less remarkable for the flavonoids than for the other antioxidant parameters analysed.

The FD powders generally presented decreased antioxidant properties as compared to the HAD ones (Figure 3). This could be explained by a higher exposure to oxidative conditions once the atmospheric pressure is restored, due to the higher porosity of FD products [65]. The high porosity of FD products allows easy access to oxygen, which may lead to higher levels of oxidation or the degradation of bioactive compounds [66,67]. These phenomena would explain the lower content of antioxidant compounds in the powders obtained by FD compared to the HAD ones. Dalmau et al. [68] reported similar results in apples subjected to FD as compared to HAD at 60 °C. Also, Rudy et al. [69] observed a decrease in total phenol content in FD blueberries. Similarly, previous studies carried out in the same laboratory on residues of various vegetables confirmed that HAD generally results in products with higher antioxidant capacity than FD ones. However, the presence of specific bioactive compounds such as carotenoids or sulforaphane is usually higher in FD products [34,70].

### 3.2. Probiotic Properties of Broccoli Waste Powders

**Figure 4:**
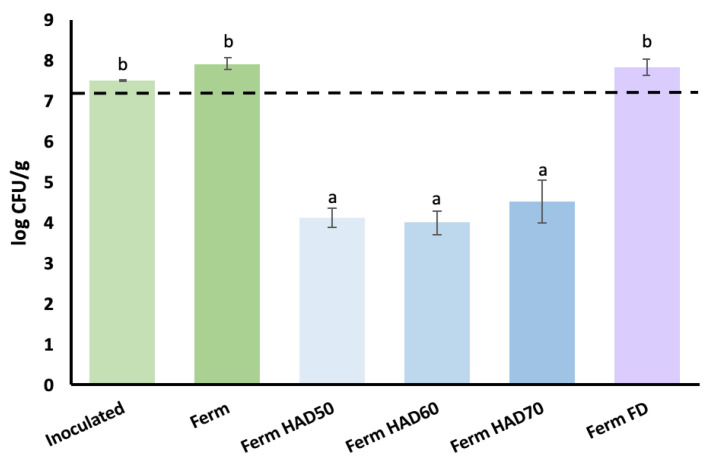
Viable counts in freshly inoculated broccoli residue after 24 h of fermentation with *L. plantarum* and in powders obtained from the fermented stems dried by hot air-drying (HAD) at 50, 60, and 70 °C and freeze-drying (FD). Error bars represent the standard deviation of four replicates from two replicas (two replicates/replica). ^a,b^ Different superscript letters indicate statistically significant differences at the 95% confidence level (*p*-value < 0.05), according to the multiple range test.

Fermentation can be explored as a pretreatment for modifying the physicochemical and antioxidant properties of a residue and the corresponding powder but also for obtaining probiotic products when probiotic strains are used as starters, on the condition that microbial viability is preserved. Figure 4 shows the results for *L. plantarum* counts in freshly inoculated broccoli stems, after 24 h of fermentation, and in the corresponding powders obtained by HAD at 50, 60, and 70 °C and FD.

To be considered probiotic, a product must contain around 10^6^–10^8^ CFU/g viable cells [71,72], with 10^7^ CFU/g generally being the minimum probiotic concentration found in commercial products [73].

The broccoli residue freshly inoculated with the starter culture had a viable cells concentration higher than 10^7^ CFU/g. After 24 h of fermentation, the fermented broccoli waste had a slightly increased concentration, confirming broccoli waste as a favourable plant matrix for *L. plantarum* growth, as reported by other authors [23,24].

The results evidenced a statistically significant impact of the drying technique on microbial viability (*p*-value < 0.05). Whereas FD allowed the preservation of microorganisms, HAD significantly reduced microbial counts below the minimum considered probiotic, regardless of the temperature applied.

These results are consistent with previous lab results [16] and other authors’ research [74]. Thus, FD is confirmed as a technique for preparing high-value stable probiotic cultures [75], as well as a modern and innovative method of drying and processing primary agricultural products [76]. However, this technique presents high equipment and operating costs and requires specialised staff [77]. Another option would be optimising HAD conditions to improve cell viability, by proposing a progressive decreasing temperature during drying, for instance.

After this first part of the study, and based on the antioxidant properties of the dried powders, it was decided to proceed with HAD at 60 °C for the second part of the research, in which pretreatments were investigated prior to HAD and FD.

### 3.3. Impact of Thermophysical and Biological Treatments on Broccoli Stem Products

#### 3.3.1. Physicochemical, Antioxidant, and Microstructural Properties of Broccoli Wastes as Affected by Thermophysical and Biological Pretreatments

The impact of the pretreatments applied on the moisture content, water activity, and soluble solids content of the broccoli wastes are shown in Table 2. As observed, the moisture content was quite similar for all samples, but slightly lower for the AUTO and MW (*p*-value < 0.05). According to the literature, autoclaving can reduce moisture content due to evaporation caused by high temperatures [78]; similarly, the MW treatment may heat up the product due to electromagnetic energy conversion into heat and evaporate part of the liquid water [79]. Similar results were obtained in a previous study on camellia seed samples subjected to microwave pretreatment at 640 W per 500 g of a sample for 5 and 8 min, where a significant reduction in moisture content was obtained [80].

As for a_w_, the fresh and pretreated samples exhibited values higher than 0.99, which imply a high susceptibility to spoilage due to enzymatic action or microbial growth [81,82].

The PAST, AUTO, and US treatments did not significantly affect the soluble solids content in the ground broccoli stems. In contrast, the application of MW implied a significant increase, whereas fermentation also reduced the soluble solids content significantly (*p*-value < 0.05). Other authors have also reported soluble solids’ increase after MW treatment [83,84], which could be related to water evaporation and subsequent solutes’ concentration due to microwave heating [85]. In addition, the interaction of biological materials with microwaves generates thermal and non-thermal effects which may cause structural changes due to the vapour explosions generated in overheating points (hotspots) as a consequence of the heterogeneous heating [86]. These points can undergo self-explosion phenomena that cause ruptures and structural modifications such as the depolymerisation of cellulose and solubilisation of lignin, releasing simpler sugars among other constituents [86,87]. Regarding FERM, the decrease in soluble sugars may be due to microorganisms using them as an energy source [88]. Similarly, Peng et al. [89] confirmed that after fermenting apple juices of different cultivars with *Lactobacillus* spp., there was a decrease in sucrose, lactose, and glucose content due to their consumption by probiotics. However, this result differs from those obtained in the first part of this study.

The antioxidant properties of the pretreated broccoli wastes are shown in Table 3, where values are given for the total phenol content (mg GAE/g_dm_), total flavonoid content (mg QE/g_dm_), and antioxidant capacity measured by the ABTS and DPPH methods (mg TE/g_dm_).

Pretreatments generally improved the antioxidant properties as compared to the fresh broccoli stems (*p*-value < 0.05). The pasteurisation and ultrasound pretreatments resulted in the highest values. US promote cavitation phenomena which cause plant tissues’ breakage and microchannels’ formation [27,28], which may lead to the release of antioxidant compounds bound to complex structures in the plant matrix. Different examples in the literature report US efficiency for increasing the antioxidant properties of vegetable matrices [90,91,92,93]. Regarding the increase in antioxidant properties after pasteurisation, similar results were reported by Urquieta-Herrero et al. [94], who obtained changunga pulp enriched in phenolic compounds after pasteurisation. In a different study about fruit juices, pasteurisation pretreatment increased the antioxidant capacity measured by the ABTS and DPPH methods [9]. This increase in antioxidant properties after pasteurisation pretreatment may be due to the temperature-induced disruption and permeabilisation of the plant matrix [95]. Also, during heat pretreatment with pasteurisation, biochemical reactions may occur, resulting in forms of antioxidant compounds with higher bioactivity [96].

Fermentation also caused an improvement in the antioxidant properties, except for the ability to scavenge the DPPH free radical. Other studies have reported an increase in antioxidant properties after fermentation with LAB in broccoli samples [34,97] or in other products such as fermented loquat juice, where higher phenols and flavonoids contents were obtained [98]. This increase could be due to the LAB, which promote the hydrolysis of complex molecules, like polyphenols and other bioactive compounds, into free and simple forms with higher bioactivities [21,23,98,99]. This is achieved through the production of certain enzymes, such as glycosidases, tannases, and esterases, which convert phenolic esters into aglycones and phenolic acids with greater antioxidant activities. Moreover, the disruption of protein–polyphenol complexes by LAB proteases may further increase phenolic content [100,101] and the LAB’s production of new antioxidant compounds [102]. Fermentation also promotes the breakdown of sugars, vitamins, and other compounds present in plant matrices [54], thus releasing phenolic constituents that would otherwise remain bound to the plant matrix. On the contrary, other authors have reported opposite results, such as those obtained in the present research in the preliminary study of fermentation and drying. Another example was found in the lactic acid fermentation of apple juice, where Wu et al. [103] reported a dramatic decrease in total phenols and flavonoids. All the previous information confirms that this pretreatment shows variability in its efficacy and could be influenced by interactions between various microbiological, enzymatic, chemical, and biochemical reactions and physical processes [56].

The MW pretreatment also caused an improvement in some antioxidant properties, which were statistically significant for the DPPH and ABTS radicals’ scavenging activities. As previously stated, the increase in antioxidant compounds may be due to the vapour explosions generated at the points where there is overheating, also known as hotspots [86,104], which occur because of the direct interaction of the food or plant material with microwaves. These hotspot explosions may release simpler phenolic compounds, which otherwise remain bound to the structure, in a more complex organisation. MW treatment has shown efficiency in lignocellulosic biomass, such as sorghum grains [105], where it was shown that the antioxidant capacity of microwave-treated samples increased significantly. The impact of MW on phenolics content and antioxidant properties may also be due to improved extractability. Álvarez et al. [106] reported that MW pretreatments intensified phenolics extraction in apple pomace and boosted anthocyanin product richness. During MW treatment, moisture is heated, leading to vaporisation and increased pressure within the vacuole; consequently, the porous cell wall ruptures, releasing phenolics from the solid and facilitating their extraction [107]. The AUTO pretreatment was the least efficient in improving the antioxidant properties of the ground broccoli stems, maybe due to the thermal decomposition of antioxidant compounds because of the temperature effect [108]. Nevertheless, the total flavonoids and ABTS antiradical activity improved with respect to the control sample in the autoclaved samples, suggesting that this thermophysical treatment may also release certain antioxidant compounds due to the high pressure and heat reached due to their effect on the tissue structure. Other authors have found a positive effect of AUTO in the phenolic content of nuts, an increase which was attributed to higher extraction yields, the formation of Maillard reaction products, and the possible release of some bound phenolic compounds due to processing conditions [109]. Nevertheless, the results were variable among nuts (pistachio, cashew, chestnut) and improved significantly when harsher autoclave conditions were applied. In addition, improvement was more significant for specific phenolic compounds and less remarkable for the antioxidant properties measured.

**Figure 5:**
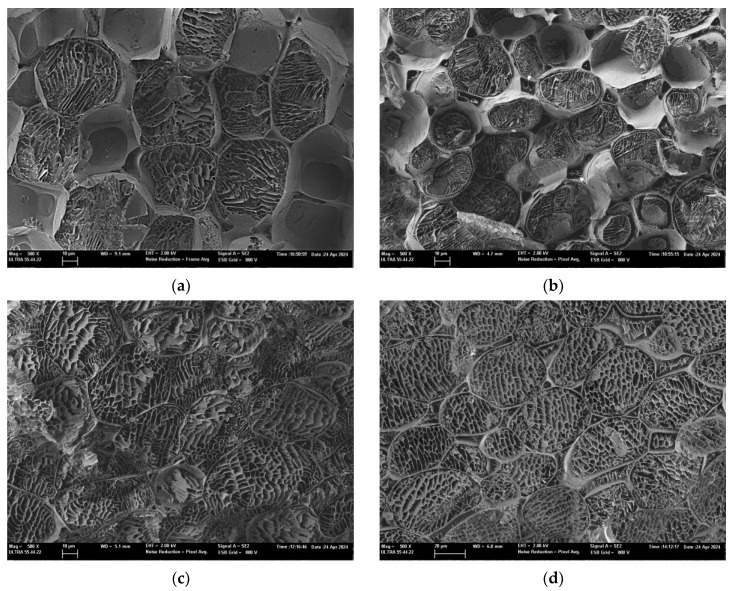
Micrographs of the pretreated residue obtained by low-temperature scanning electron microscopy (cryo-SEM) at 500× magnification (bar = 10 microns). (**a**) Ground broccoli residue; (**b**) ultrasonically pretreated broccoli residue; (**c**) fermented broccoli residue; (**d**) autoclaved broccoli residue. Arrows in (**b**) indicate intercellular spaces.

Microscopical images of the pretreated broccoli residue were obtained to verify the impact of the pretreatments on the plant tissue structure. Micrographs of selected pretreatments were obtained by scanning electron microscopy at low temperatures (cryo-SEM). Non-pretreated (a), fermented (b), ultrasonicated (c), and autoclaved (d) samples are shown in Figure 5.

The non-pretreated ground broccoli (Figure 5a) shows classic parenchymatic tissue in which large rounded cells with cell walls can be identified. The intercellular spaces or pores appear empty, with no reticulum due to liquid phase release, which is a sign of tissue integrity. In contrast, in the micrograph corresponding to the ultrasonicated residue (Figure 5b), it can be seen that some intercellular spaces or pores are occupied by fluid coming from inside the cells, which evidences cell walls’ and membranes’ permeabilisation due to cavitation phenomena [110]. Kumar et al. [111] confirmed that the generation and collapse of cavitation bubbles induces turbulence within the fluid, resulting in cell walls’ and membranes’ rupture releasing active compounds of interest. Also, we can observe (Figure 5b) the appearance of larger intercellular spaces and membrane separations from the corresponding cell walls due to mechanical vibration [112]. These structural changes, together with increased porosity, may facilitate the extraction of components from plant matrices [113]. These microscopic observations are consistent with the improvement in the antioxidant properties observed for the US pretreated samples.

Figure 5c shows a micrograph of the fermented tissue, where a significant loss of cell integrity and compartmentalisation of cell structures can be observed. While some cells maintain a defined shape, others show manifest signs of decompartmentalisation, such as an irregular shape and unstructured and degraded walls, suggesting a certain depolymerisation of the cell walls. Additionally, the liquid phase is present throughout all the tissue, indicating a greater loss of compartmentalisation and release of cellular compounds. This breakdown of the plant matrix is due to the ability of lactic acid bacteria, such as *L. plantarum*, to metabolise and transform complex and indigestible proteins, cellulose, and other substances into simpler ones [54]. These microscopic observations are consistent with the improved antioxidant properties obtained for the fermented residue, since the observed decompartmentalisation and depolymerisation contribute to the release of compounds with antioxidant activity (Table 3).

Finally, in the case of the residue which underwent autoclave pretreatment (Figure 5d), the differences with respect to the non-pretreated control (Figure 5a) are not as evident, which agrees with the less significant improvement in the antioxidant properties observed in this case. However, this high temperature and pressure treatment would also have caused some permeabilisation of membranes, as reticulated spaces are observed inside the pores and intercellular spaces, indicating the presence of the liquid phase both inside and outside the cells. In addition, the image obtained suggests less rigidity in the cell walls, which is consistent with some degradation or solubilisation of the structures.

#### 3.3.2. Impact of Pretreatments on the Properties of Powdered Broccoli Stem Products

Table 4 shows the physicochemical properties of the powders obtained by the HAD and FD of the fresh and pretreated broccoli stems.

As expected, the powders showed significantly lower a_w_ and x_w_ values (*p*-value < 0.05) than their non-dehydrated counterparts (Table 2). These values fall within the range considered adequate to ensure the stability of such products (a_w_ between 0.20 and 0.35; x_w_ < 0.1 g_w_/g_total_), effectively preventing the growth of spoilage bacteria and extending the shelf life of the resulting powders [48,114]. FD resulted in powders with generally lower a_w_ values (*p*-value < 0.05), probably due to an enhanced efficiency of water removal achieved through the prior freezing of the ground residue, followed by sublimation and desorption [115], thus leading to the formation of porous channels as the ice sublimates.

Statistically significant differences in moisture content values (*p*-value < 0.05) were observed, with higher values in the HAD powders, indicating differences in water removal mechanisms depending on the drying technique used. During convective drying, water is transferred from inside the product to the product–air interface and then removed from the surface in the vapour state, leading to tissue shrinkage that can limit further moisture transfer [52,116]. This process results in the formation of a surface layer with increased resistance, which results in a dry surface, while the inner side remains moist [52,77]. This phenomenon is more pronounced as the drying rate increases [11,117]. As evidenced in previous studies, fermentation may accelerate surface water removal and contribute to case-hardening incidence [34]. The major impact of drying on fermented residues is related to structural changes, including the breakdown of the plant cell wall and pore formation [34,117]. This could explain why the moisture content of powders obtained through fermentation followed by hot air-drying at 60 °C (FERM HAD60) was the highest.

Among the HAD60 samples, the US HAD60 and MW HAD60 ones reached lower moisture contents compared to the non-pretreated powders, as well as significantly lower a_w_ values (*p*-value < 0.05) than those subjected to other pretreatments. The moisture content of the US HAD60 powders was similar to the respective FD powders. Increased water availability in the liquid phase of the tissue, or reduced mass transfer resistance in the tissue due to membrane and cell wall permeabilisation caused by cavitation, could explain this. Additionally, the US HAD60 powders had significantly lower a_w_ values (*p*-value < 0.05) than the US FD powders, further confirming, along with the moisture data, the contribution of the pretreatment to drying efficiency. Most of the HAD powders exhibited lower x_ss_ values than their respective FD ones. This was confirmed by the multifactorial ANOVA considering the pretreatment and dehydration techniques as factors, since the dehydration technique resulted in being significant (*p*-value < 0.05). This could be due to the FD process, which facilitates extraction due to ice crystals’ formation, which causes disruption [115,118]. In addition, FD produces a more porous structure [14], which facilitates milling, and generally yields a smaller particle size.

**Figure 6:**
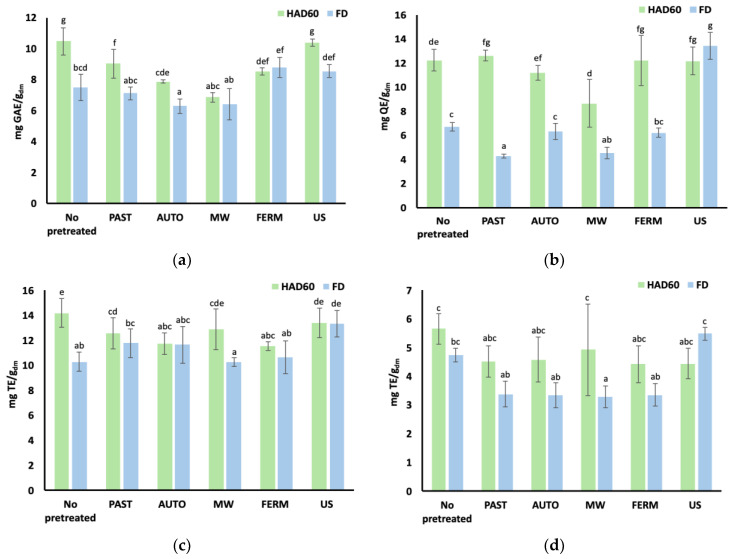
Antioxidant properties of pretreated and non-pretreated powders obtained by different dehydration techniques: (**a**) Total phenol content expressed as mg gallic acid equivalent (GAE) per gram of dry matter. (**b**) Total flavonoid content expressed as mg quercetin equivalent (QE) per gram of dry matter. (**c**,**d**) ABTS and DPPH antioxidant capacity, respectively, expressed in mg trolox equivalent (TE) per gram of dry matter. PAST: pasteurisation; AUTO: autoclaving; MW: microwaves; FERM: fermentation; US: ultrasounds. Error bars represent standard deviation of six replicates from two replicas (3 replicates/replica). ^a–g^ Different superscripts indicate statistically significant differences at 95% confidence level (*p*-value < 0.05), according to multiple range test.

The antioxidant properties of the powdered products obtained are shown in Figure 6, where the total phenols (mg GAE/g_dm_), total flavonoids (mg QE/g_dm_), and overall antioxidant activity measured by the ABTS and DPPH methods (mg TE/g_dm_) are shown.

Dehydration caused an improvement in the antioxidant properties of the broccoli residue (*p*-value < 0.05), as compared to the non-dehydrated stems (Table 3). This increase might be attributed to the various processing steps, including grinding, pretreatment, drying, and milling, which likely contributed to the breakdown of the plant matrix, facilitating the release of bioactive compounds or triggering other biochemical changes that improved the antioxidant properties [119]. Notably, the antioxidant properties were generally higher in powders obtained by HAD at 60 °C than by FD. This difference may be due to Maillard reactions, which occur during HAD [120], leading to the formation of antioxidant compounds. Such reactions are unlikely during FD because of the low temperatures involved. The role of Maillard reactions in enhancing antioxidant compounds was studied by Somjai et al. [121], who observed increased antioxidant capacity in Chinese lemon peels dried at 60 °C, as measured by ABTS and DPPH methods. In addition to Maillard reactions, other biochemical reactions favoured by heat could generate more active forms of antioxidant compounds; trans–cis isomerisation [63], for instance, inhibits pro-oxidant enzymes’ activity [122] or activates enzymes that hydrolyse compounds into more active forms, such as in the hydrolysis of glucosinolates into isothiocyanates by myrosinase action [123]. These factors collectively could explain the observed increase in the antioxidant properties.

Multifactor ANOVA analysis confirmed that both the pretreatment and the drying method significantly influenced (*p*-value < 0.05) the antioxidant properties of the powders. Likewise, the interaction between the pretreatment used and the type of dehydration method was also significant (*p*-value < 0.05) for all the antioxidant properties analysed. This reveals that the impact of the drying technique on antioxidant properties varies depending on the pretreatment applied, which is likely influenced by the different conditions that occur in each of the dehydration techniques [124].

Regarding total phenol content (Figure 6a), none of the pretreatments improved total phenol levels in the products subjected to HAD. However, among the FD powders, pretreatments with US, and especially FERM, led to significant improvements. This suggests that the antioxidant compounds released or generated during fermentation and ultrasound pretreatment may be more susceptible to degradation by HAD or are better extracted due to the physical characteristics of FD powders [17,54,111]. In both cases, these pretreatments, along with pasteurisation, contributed to higher phenol content in both the HAD and FD powders. Conversely, the pasteurised residue, which initially had the highest total phenol content before dehydration, resulted in powders with phenolic levels comparable to those without pretreatment.

In terms of flavonoids, the HAD powders showed a significant improvement (*p*-value < 0.05) compared to non-pretreated powders in all cases except for the MW and AUTO pretreatments, where no significant differences were observed (*p*-value > 0.05). Similarly, Vargas et al. [125] found that the HAD of broccoli, kale, and spinach led to a temperature-dependent release of flavonoids bound in the plant matrix. This release may also be favoured by pretreatments such as FERM and US, which cause direct damage to the plant matrix [54,111], explaining the increase in flavonoids for these pretreatments (Figure 6b). Similarly, the FD powders were significantly (*p*-value < 0.05) enriched in flavonoids when undergoing US pretreatment, likely due to the increased damage to the plant tissue architecture, as previously mentioned.

In terms of antioxidant capacity, different results were obtained depending on the method used. No improvement was obtained according to the DPPH radical method (Figure 6d). However, the ABTS radical method showed enhanced antioxidant capacity in the powders pretreated with US and further FD (Figure 6c). This increase in the extraction of antioxidant compounds could be attributed to the cavitation effect induced in the broccoli residue by high ultrasound frequencies, as well as by the formation of channels in the plant matrix after the sublimation of ice crystals [110,118]. These findings confirm that the US pretreatment is the one that allowed us to obtain FD powders with better antioxidant properties.

With regard to powders obtained from the broccoli residues subjected to a fermentation pretratement, viable cell counts were obtained for the freshly inoculated broccoli stems, after 24 h of fermentation with *L. plantarum*, and following dehydration by HAD and FD. As mentioned in the preliminary study, a product must contain a viable cell concentration of approximately 10^6^–10^7^ CFU/g to be considered probiotic [71,72]. For the freshly inoculated broccoli stem, the count was at a concentration of 10^7^ CFU/g, so it could be considered potentially probiotic. After 24 h of fermentation, there was a significant increase (*p*-value < 0.05) in the viable cell count, confirming that broccoli is a suitable plant matrix for the growth of probiotic microorganisms [23,80].

After subjecting the fermented broccoli stems to HAD in the pilot plant’s convective dryer, and unlike in preliminary studies using benchtop dryers, no viable counts were observed in the seeded plates (counts < 10^4^ CFU/g). This result is likely due to the negative impact of the drying temperature or to an excessively prolonged treatment. Previous studies have shown that convective drying at temperatures above 40 °C can reduce the viability of probiotic bacteria [126], but other research on similar waste products demonstrated that viable microorganisms can be preserved in powders dried at 50, 60, and 70 °C [34]. Therefore, it becomes crucial to optimise the drying time to not extend the duration of the falling drying rate period, during which the product’s temperature can exceed the probiotic tolerance threshold. In the present study, all pretreated products were dried under the same temperature and time conditions for comparison purposes. This decision could have been detrimental for the viability of the probiotics, in the light of the results obtained for the microorganism viability after drying. In contrast, the FD powders exhibited fewer viable cells after dehydration (*p*-value < 0.05), but the powders still maintained a potentially probiotic count of 10^6^ CFU/g. FD is generally preferred for preserving microbial viability due to its use of low temperatures and absence of oxygen, making it a suitable technique for producing high-value, stable probiotic cultures [75].

Table 5 displays the powders’ phenolic profiles obtained from chromatographic analyses (HPLC). The content of specific phenolic compounds may vary depending on the pretreatment and the dehydration method used. After comparing with standard absorption spectra and retention times, none of the following phenolics were identified in the powder extracts: 4-Hydroxibezoic acid, epicatechin, vanillin, apigenin-7-glucoside, trans-cinnamic acid, naringenin, and kaempherol.

To facilitate the results’ interpretation, phenolic constituents were gathered in groups so that the total hydroxycinnamic acids, total hydroxybenzoic acids, and total flavonoids were presented, besides the total phenolic content. The HAD powders not subjected to pretreatments showed higher phenolic contents than the FD ones, which is consistent with the antioxidant properties exhibited (Figure 6). However, when pretreatments were applied, the values in the FD powders were generally higher than in their HAD counterparts. On the one hand, this could be explained by the facilitated extraction of bioactive compounds from FD products due to their characteristic porous structure, coupled with the effect of the pretreatments on the cellular tissue, which would have favoured the release of phenolic compounds from the vegetal matrix. On the other hand, release of phenolic constituents during pretreatments followed by HAD could have resulted in an increased exposure of these compounds to high temperatures and oxidative conditions, thus reducing their concentration in the pretreated HAD samples.

In the fermented samples (both FD and HAD), a decrease in the glycosides analysed, together with an increase in aglycone forms, was registered, a result which is in accordance with Lee et al. [127], who reported the conversion of flavonoid glycosides to flavonols in silkworm thorn leaves due to fermentation with *Lactobacillus plantarum*. On the other hand, phenolic acids’ degradation by lactic acid bacteria is an important mechanism for the detoxification of these compounds. According to the literature, *Lactobacillus* spp. exhibit the strain-specific metabolism of phenolic acids including hydroxybenzoic acids, hydroxycinnamic acids, and hydroxycinnamic acid derivatives; particularly, Filannino et al. [128] evidenced that the metabolism of phenolic acids by *L. plantarum* is strain-specific. In this work, fermented samples were amongst the ones which presented higher phenolic acids content.

As for flavonoids, rutin was only identified in fermented powders (FERM HAD60 and FERM FD), exhibiting a significantly higher value for the HAD powder than for the FD one (4.89 ± 0.03 vs. 1.7 ± 0.4 mg/100 g_dm_, respectively). The fact that this compound was only present in the fermented samples suggests that *L. plantarum* might be involved in the release of this compound. On the other hand, quercetin was only found in the non-pretreated powders (HAD60 and FD), thus suggesting that pretreatments might have a negative impact on it.

Hydroxycinnamic acids act as powerful antioxidants in dried broccoli [129,130]. As observed in Table 5, the value of individual hydroxycinnamic acids varied significantly among the different pretreatments. However, powders obtained by the FD method presented higher contents of these compounds than their HAD counterparts, except for PAST-pretreated samples. Since hydroxycinnamic acids are heat-sensitive, FD conditions, i.e., lower temperatures and reduced oxygen, could have prevented the degradation found in the HAD samples. This fact has been previously reported in other studies about dried broccoli, in which hydroxycinnamic acids (such as ferulic acid, caffeic acid, or coumaric acid) were affected by the heat and oxidative conditions of convective drying [125,129]. Finally, gallic acid was the only hydroxybenzoic acid identified in the broccoli powders. It was present in the unpretreated HAD samples and in powders obtained from the pasteurised samples, both with FD or HAD.

## 4. Conclusions

Biological and thermophysical pretreatments have an impact on tissue structure, thus releasing bioactive compounds which contribute to antioxidant properties. This study on biological and thermophysical pretreatments applied to fresh ground tissue confirmed that these pretreatments can successfully contribute to improving the antioxidant properties of ground broccoli stems. Particularly, ultrasound application exhibited the most remarkable increase, whereas autoclaving and microwaving led to less evident improvements. Changes in the antioxidant properties of the broccoli wastes were related to the changes observed in the microstructure, such as permeabilisation of membranes and cell walls, and loss of cell compartmentalisation. Treatments such as ultrasounds, microwaves, or fermentation could also have led to the release of simpler phenolic constituents initially bound to the structure as part of more complex forms. In contrast, the thermal degradation of some bioactive constituents could also have occurred in treatments such as the autoclave or microwave treatments. Interestingly, dehydration applied to the pretreated ground broccoli stems improved their antioxidant properties, especially in the HAD ones, but not as much as in powders obtained from the non-pretreated broccoli wastes. The powders obtained from the ultrasonicated ground broccoli stems were the only pretreated powders which exhibited improved antioxidant properties, and this was obtained only for some antioxidant parameters. In general, the HAD powders showed better antioxidant properties than the freeze-dried ones. The probiotic properties of the powders were not maintained when scaling up to the pilot plant air drier, although this could be attributed to the excessive duration of the treatment. In contrast, the freeze-dried products maintained their probiotic properties.

In conclusion, thermophysical and biological pretreatments might be proposed to enhance the antioxidant attributes of broccoli wastes but not necessarily that of dried powdered products. Further research should focus on the drying kinetics and duration of this stage after pretreatments to better adjust drying parameters.

Once refined, these processes could be implemented at an industrial scale to support sustainable practises and circular economy principles. By transforming the entire broccoli stem into functional powders, this study contributes to reducing food waste and promotes the reintroduction of these residues into the food supply chain as valuable ingredients. Being rich in antioxidant compounds, these products have market potential for food fortification. The powders could be effectively incorporated into a variety of processed products such as baked goods, pasta, sauces, meat products or analogues, and smoothies, among others, offering nutritional enhancement while supporting sustainable production practises.

## Figures and Tables

**Table 1:** Water content (x_w_), water activity (a_w_), and total soluble solids content (x_ss_) of non-dehydrated samples (fresh, fermented) and dehydrated (by hot air-drying at 50, 60, or 70 °C (HAD) or freeze-drying (FD)) powders obtained from fermented (Ferm HAD, Ferm FD) and unfermented (HAD, FD) broccoli stems. Mean ± standard deviation of three replicates.

Treatment	x_w_ (g_w_/g_total_)	a_w_	x_ss_ (g_ss_/g_dm_)
FRESH	0.9163 ± 0.0011 ^f^	0.9932 ± 0.0014 ^g^	0.69 ± 0.04 ^de^
FERM	0.9128 ± 0.0007 ^f^	0.9920 ± 0.0012 ^g^	0.654 ± 0.008 ^bcd^
HAD 50	0.054 ± 0.005 ^a^	0.23 ± 0.02 ^c^	0.600 ± 0.016 ^a^
HAD 60	0.054 ± 0.002 ^a^	0.215 ± 0.011 ^bc^	0.599 ± 0.016 ^a^
HAD 70	0.055 ± 0.003 ^a^	0.249 ± 0.006 ^d^	0.61 ± 0.00 ^ab^
Ferm HAD 50	0.093 ± 0.012 ^c^	0.200 ± 0.006 ^ab^	0.673 ± 0.017 ^cde^
Ferm HAD 60	0.1075 ± 0.0014 ^de^	0.193 ± 0.012 ^a^	0.637 ± 0.017 ^abc^
Ferm HAD 70	0.0991 ± 0.0010 ^cd^	0.2110 ± 0.0016 ^bc^	0.64 ± 0.00 ^abc^
FD	0.075 ± 0.011 ^b^	0.280 ± 0.013 ^e^	0.72 ± 0.00 ^e^
Ferm FD	0.115 ± 0.006 ^e^	0.330 ± 0.013 ^f^	0.70 ± 0.04 ^de^

^a–g^ Different superscript letters in the same column indicate statistically significant differences at the 95% confidence level (*p*-value < 0.05), according to the multiple range test.

**Table 2:** Effect of thermophysical and biological pretreatments on the moisture content (x_w_), water activity (a_w_), and total soluble solids content (x_ss_) of broccoli stems. PAST: pasteurisation; AUTO: autoclaving; MW: microwaves; FERM: fermentation; US: ultrasounds. Mean ± standard deviation of three replicates.

Treatment	x_w_ (g_w_/g_total_)	a_w_	x_ss_ (g_ss_/g_dm_)
FRESH	0.934 ± 0.004 ^c^	0.9910 ± 0.0009 ^ab^	0.66 ± 0.04 ^b^
PAST	0.934 ± 0.002 ^c^	0.9940 ± 0.0013 ^c^	0.65 ± 5 0.018 ^b^
AUTO	0.928 ± 0.004 ^b^	0.9931 ± 0.0009 ^bc^	0.66 ± 0.00 ^b^
MW	0.913 ± 0.004 ^a^	0.9947 ± 0.0009 ^c^	0.678 ± 0.006 ^c^
FERM	0.938 ± 0.003 ^c^	0.990 ± 0.003 ^a^	0.55 ± 0.08 ^a^
US	0.93 ± 0.00 ^c^	0.9948 ± 0.0014 ^c^	0.669 ± 0.011 ^b^

^a–c^ Different superscript letters in the same column indicate statistically significant differences at the 95% confidence level (*p*-value < 0.05), according to the multiple range test.

**Table 3:** Effect of different pretreatments on total phenol content (mg GAE/g_dm_), total flavonoid content (mg QE/g_dm_), and antioxidant capacity by ABTS and DPPH (mg TE/g_dm_) of fresh broccoli stems. PAST: pasteurisation; AUTO: autoclaving; MW: microwaves; FERM: fermentation; US: ultrasounds. Mean ± standard deviation of six replicates from two replicas (3 replicates/replica).

Treatment	Total Phenols (mg GAE/g_dm_)	Total Flavonoids (mg QE/g_dm_)	ABTS (mg TE/g_dm_)	DPPH (mg TE/g_dm_)
FRESH	4.2 ± 0.2 ^a^	2.05 ± 0.17 ^a^	5.8 ± 0.2 ^a^	1.01 ± 0.10 ^a^
PAST	6.1 ± 0.004 ^c^	2.58 ± 0.18 ^b^	9.5 ± 0.4 ^c^	3.5 ± 0.7 ^b^
AUTO	4.05 ± 0.11 ^a^	4.1 ± 0.2 ^c^	7.56 ± 0.14 ^b^	1.9 ± 0.2 ^a^
MW	4.8 ± 0.3 ^ab^	2.02 ± 0.11 ^a^	7.6 ± 0.3 ^b^	3.87 ± 0.14 ^b^
FERM	5.3 ± 0.3 ^bc^	2.9 ± 0.3 ^b^	7.9 ± 0.4 ^b^	1.43 ± 0.07 ^a^
US	5.79 ± 0.18 ^c^	4.88 ± 0.12 ^d^	9.2 ± 0.7 ^c^	3.7 ± 0.2 ^b^

^a–c^ Different superscript letters in the same column indicate statistically significant differences at the 95% confidence level (*p*-value < 0.05), according to the multiple range test.

**Table 4:** Values for moisture content (x_w_), water activity (a_w_), and total soluble solids content (x_ss_) of powders obtained by hot air-drying or freeze-drying of fresh and pretreated broccoli stems. PAST: pasteurisation; AUTO: autoclaving; MW: microwaves; FERM: fermentation; US: ultrasounds. HAD: hot air-drying; FD: freeze-drying. Mean ± standard deviation of three replicates.

Treatment	x_w_ (g_w_/g_total_)	a_w_	x_ss_ (g_ss_/g_dm_)
HAD60	0.059 ± 0.002 ^de^	0.243 ± 0.005 ^b^	0.670 ± 0.016 ^bcdef^
PAST HAD60	0.068 ± 0.002 ^f^	0.307 ± 0.005 ^h^	0.655 ± 0.016 ^abcd^
AUTO HAD60	0.060 ± 0.003 ^e^	0.305 ± 0.005 ^h^	0.64 ± 0.02 ^abc^
MW HAD60	0.0493 ± 0.0011 ^c^	0.268 ± 0.006 ^de^	0.630 ± 0.011 ^a^
FERM HAD60	0.115 ± 0.003 ^g^	0.302 ± 0.004 ^gh^	0.631 ± 0.011 ^a^
US HAD60	0.0520 ± 0.0014 ^cd^	0.26 ± 0.008 ^cd^	0.688 ± 0.016 ^def^
FD	0.033 ± 0.005 ^a^	0.261 ± 0.008 ^cd^	0.678 ± 0.011 ^cdef^
PAST FD	0.059 ± 0.004 ^de^	0.2495 ± 0.0017 ^bc^	0.663 ± 0.016 ^abcde^
AUTO FD	0.046 ± 0.004 ^bc^	0.281 ± 0.006 ^ef^	0.68 ± 0.02 ^def^
MW FD	0.042 ± 0.003 ^b^	0.260 ± 0.007 ^cd^	0.70 ± 0.02 ^f^
FERM FD	0.057 ± 0.010 ^de^	0.208 ± 0.005 ^a^	0.691 ± 0.005 ^ef^
US FD	0.048 ± 0.002 ^bc^	0.290 ± 0.008 ^fg^	0.64 ± 0.03 ^ab^

^a–h^ Different superscript letters in the same column indicate statistically significant differences at the 95% confidence level (*p*-value < 0.05), according to the multiple range test.

**Table 5:** Phenolic content (mg/100 g_dm_) of powders obtained by hot air-drying or freeze-drying from fresh and pretreated broccoli stems. PAST: pasteurisation; AUTO: autoclaving; MW: microwaves; FERM: fermentation; US: ultrasounds. HAD: hot air-drying; FD: freeze-drying. Mean ± standard deviation of three replicates. n.d. not detected. ^a–g^ Different superscripts indicate statistically significant differences at 95% confidence level (*p*-value < 0.05), according to multiple range test.

	Phenolic Compounds (mg/100 g_dm_)																			
	Hydroxycinamic Acids							Hydroxybenzoic Acids			Flavonoids									Total
	Sinapic Acid	Caffeic Acid	*p*-Coumaric Acid	Ferulic Acid	4-*O*-Caffeoyl-Quinic	*Trans*-Cinnamic Acid	Total	Gallic Acid	4-Hydroxibezoic Acid	Total	Epicatechin	Quercitin 3-Glucoside	Rutin	Quercitrin	Naringenin	Apigenin-7-Glucoside	Quercetin	Kaempherol	Total	
HAD60	0.928 ± 0.014 ^f^	0.609 ± 0.006 ^a^	0.406 ± 0.002 ^a^	0.91 ± 0.11 ^c^	2.1 ± 0.2 ^c^	n.d.	5.0 ± 0.3 ^cd^	3.13 ± 0.08 ^a^	n.d.	3.13 ± 0.08 ^a^	n.d.	2.5 ± 0.5 ^d^	n.d.	0.71 ± 0.08 ^b^	n.d.	n.d.	1.640 ± 0.008 ^b^	n.d.	4.9 ± 0.6 ^e^	13.03 ± 0.23 ^f^
PAST HAD60	0.64 ± 0.02 ^ab^	3.25 ± 0.13 ^de^	0.570 ± 0.016 ^cd^	0.75 ± 0.04 ^b^	0.46 ± 0.03 ^a^	n.d.	5.7 ± 0.2 ^de^	5.3 ± 1.7 ^a^	n.d.	5.3 ± 1.7 ^a^	n.d.	1.28 ± 0.09 ^abc^	n.d.	n.d.	n.d.	n.d.	n.d.	n.d.	1.28 ± 0.09 ^ab^	12.3 ± 1.5 ^f^
AUTO HAD60	0.79 ± 0.03 ^bcdef^	1.4 ± 0.3 ^b^	n.d.	n.d.	1.148 ± 0.016 ^b^	n.d.	3.0 ±0.5 ^a^	n.d.	n.d.	n.d.	n.d.	1.8 ± 0.2 ^cd^	n.d.	n.d.	n.d.	n.d.	n.d.	n.d.	1.8 ± 0.2 ^bc^	4.9 ± 0.3 ^a^
MW HAD60	0.74 ± 0.08 ^bcde^	1.64 ± 0.07 ^bc^	0.53 ± 0.05 ^c^	n.d.	0.78 ± 0.02 ^ab^	n.d.	3.64 ± 0.14 ^ab^	n.d.	n.d.	n.d.	n.d.	1.58 ± 0.05 ^bc^	n.d.	0.523 ± 0.013 ^a^	n.d.	n.d.	n.d.	n.d.	2.10 ± 0.03 ^c^	5.74 ± 0.16 ^ab^
FERM HAD60	0.95 ± 0.02 ^f^	0.59 ± 0.02 ^a^	n.d.	1.03 ± 0.06 ^d^	0.55 ± 0.02 ^a^	n.d.	3.12 ± 0.11 ^a^	n.d.	n.d.	n.d.	n.d.	1.624 ± 0.016 ^bc^	4.89 ± 0.03 ^b^	0.674 ± 0.015 ^b^	n.d.	n.d.	n.d.	n.d.	7.18 ± 0.02 ^f^	10.30 ± 0.14 ^de^
US HAD60	0.68 ± 0.05 ^abcd^	3.30 ± 0.17 ^e^	0.466 ± 0.013 ^b^	n.d.	0.556 ± 0.016 ^a^	n.d.	4.8 ± 0.3 ^c^	n.d.	n.d.	n.d.	n.d.	0.73 ± 0.05 ^a^	n.d.	n.d.	n.d.	n.d.	n.d.	n.d.	0.73 ± 0.05 ^a^	5.6 ± 0.3 ^a^
FD	0.56 ± 0.03 ^a^	0.634 ± 0.005 ^a^	n.d.	0.600 ± 0.006 ^a^	4.59 ± 0.17 ^d^	n.d.	6.38 ± 0.16 ^e^	n.d.	n.d.	n.d.	n.d.	0.8252 ± 0.0016 ^a^	n.d.	1.05 ± 0.10 ^c^	n.d.	n.d.	1.613 ± 0.002 ^a^	n.d.	3.40 ± 0.10 ^d^	9.9 ± 0.2 ^d^
PAST FD	0.7 ± 0.3 ^bcde^	2.0 ± 0.4 ^c^	n.d.	0.60 ± 0.03 ^a^	n.d.	n.d.	3.4 ± 0.7 ^ab^	10 ± 3 ^b^	n.d.	10 ± 3 ^b^	n.d.	1.04 ± 0.03 ^ab^	n.d.	0.53 ± 0.09 ^a^	n.d.	n.d.	n.d.	n.d.	1.58 ± 0.05 ^bc^	15 ± 3 ^ef^
AUTO FD	0.676 ± 0.012 ^abc^	0.834 ± 0.006 ^a^	n.d.	1.615 ± 0.016 ^f^	1.172 ± 0.014 ^b^	n.d.	4.29 ± 0.04 ^bc^	n.d.	n.d.	n.d.	n.d.	3.4 ± 1.2 ^e^	n.d.	n.d.	n.d.	n.d.	n.d.	n.d.	3.4 ± 1.2 ^d^	7.3 ± 1.4 ^bc^
MW FD	0.88 ± 0.04 ^ef^	2.9 ± 0.5 ^d^	0.58 ± 0.02 ^d^	1.29 ± 0.04 ^e^	1.10 ± 0.05 ^b^	n.d.	6.1 ± 0.8 ^e^	n.d.	n.d.	n.d.	n.d.	1.60 ± 0.12 ^bc^	n.d.	0.434 ± 0.013 ^a^	n.d.	n.d.	n.d.	n.d.	2.03 ± 0.11 ^c^	8.1 ± 0.8 ^c^
FERM FD	0.84 ± 0.10 ^def^	1.91 ± 0.03 ^c^	0.409 ± 0.005 ^a^	0.881 ± 0.006 ^c^	8.5 ± 0.9 ^e^	n.d.	13.0 ± 0.9 ^g^	n.d.	n.d.	n.d.	n.d.	1.85 ± 0.05 ^cd^	1.7 ± 0.4 ^a^	0.48 ± 0.05 ^a^	n.d.	n.d.	n.d.	n.d.	3.82 ± 0.08 ^d^	16.8 ± 0.8 ^g^
US FD	0.80 ± 0.02 ^cdef^	4.92 ± 0.06 ^e^	0.609 ± 0.004 ^d^	1.65 ± 0.05 ^f^	0.83 ± 0.05 ^ab^	n.d.	8.6 ± 0.4 ^f^	n.d.	n.d.	n.d.	n.d.	1.70 ± 0.06 ^bc^	n.d.	2.015 ± 0.013 ^d^	n.d.	n.d.	n.d.	n.d.	3.72 ± 0.07 ^d^	12.3 ± 0.4 ^f^

## Data Availability

The original contributions presented in the study are included in the article, further inquiries can be directed to the corresponding author.

## References

[1] Rajković M.B., Popović M.D., Milinčić D., Zdravković M. (2020). Circular economy in food industry. Zaštita Mater..

[2] FAO, WHO (2019). Sustainable Healthy Diets—Guiding Principles.

[3] Sharma P., Gaur V.K., Sirohi R., Varjani S., Hyoun Kim S., Wong J.W.C. (2021). Sustainable processing of food waste for production of bio-based products for circular bioeconomy. Bioresour. Technol..

[4] Li H., Xia Y., Liu H.-Y., Guo H., He X.-Q., Liu Y., Wu D.-T., Mai Y.-H., Li H.-B., Zou L. (2022). Nutritional values, beneficial effects, and food applications of broccoli (*Brassica oleracea* var. *italica Plenck*). Trends Food Sci. Technol..

[5] (2022). FAOSTAT. https://www.fao.org/faostat/en/#data/QCL.

[6] Kaparapu J., Pragada P.M., Narasimha M., Geddada R. (2020). Fruits and Vegetables and its Nutritional Benefits. Functional Foods and Nutraceuticals.

[7] Ganesh K.S., Sridhar A., Vishali S. (2022). Utilization of fruit and vegetable waste to produce value-added products: Conventional utilization and emerging opportunities-A review. Chemosphere.

[8] Núñez-Gómez V., González-Barrio R., Baenas N., Moreno D.A., Periago M.J. (2022). Dietary-Fibre-Rich Fractions Isolated from Broccoli Stalks as a Potential Functional Ingredient with Phenolic Compounds and Glucosinolates. Int. J. Mol. Sci..

[9] Mandha J., Shumoy H., Matemu A.O., Raes K. (2023). Characterization of fruit juices and effect of pasteurization and storage conditions on their microbial, physicochemical, and nutritional quality. Food Biosci..

[10] Bas-Bellver C., Barrera C., Betoret N., Seguí L. (2022). Impact of Disruption and Drying Conditions on Physicochemical, Functional and Antioxidant Properties of Powdered Ingredients Obtained from Brassica Vegetable By-Products. Foods.

[11] Bas-Bellver C., Barrera C., Betoret N., Seguí L. (2020). Turning Agri-Food Cooperative Vegetable Residues into Functional Powdered Ingredients for the Food Industry. Sustainability.

[12] Bas-Bellver C., Barrera C., Betoret N., Seguí L., Harasym J. (2024). IV-Range Carrot Waste Flour Enhances Nutritional and Functional Properties of Rice-Based Gluten-Free Muffins. Foods.

[13] Singh L., Kaur S., Aggarwal P. (2022). Techno and bio functional characterization of industrial potato waste for formulation of phytonutrients rich snack product. Food Biosci..

[14] Yao J., Chen W., Fan K. (2023). Novel Efficient Physical Technologies for Enhancing Freeze Drying of Fruits and Vegetables: A Review. Foods.

[15] Miletić N., Mitrović O., Popović B., Nedović V., Zlatković B., Kandić M. (2013). Polyphenolic Content and Antioxidant Capacity in Fruits of Plum (*Prunus domestica* L.) Cultivars “Valjevka” and “Mildora” as Influenced by Air Drying. J. Food Qual..

[16] Bas-Bellver C., Barrera C., Betoret N., Seguí L. (2023). Physicochemical, Technological and Functional Properties of Upcycled Vegetable Waste Ingredients as Affected by Processing and Storage. Plant Foods Hum. Nutr..

[17] Xu Y., Xiao Y., Lagnika C., Li D., Liu C., Jiang N., Song J., Zhang M. (2020). A comparative evaluation of nutritional properties, antioxidant capacity and physical characteristics of cabbage (*Brassica oleracea* var. *Capitate* var L.) subjected to different drying methods. Food Chem..

[18] Bassey E.J., Cheng J.H., Sun D.W. (2021). Novel nonthermal and thermal pretreatments for enhancing drying performance and improving quality of fruits and vegetables. Trends Food Sci. Technol..

[19] Mohammed H.H., Tola Y.B., Taye A.H., Abdisa Z.K. (2022). Effect of pretreatments and solar tunnel dryer zones on functional properties, proximate composition, and bioactive components of pumpkin (*Cucurbita maxima*) pulp powder. Heliyon.

[20] Ummat V., Sivagnanam S.P., Rajauria G., O’Donnell C., Tiwari B.K. (2021). Advances in pre-treatment techniques and green extraction technologies for bioactives from seaweeds. Trends Food Sci. Technol..

[21] Li Z., Teng J., Lyu Y., Hu X., Zhao Y., Wang M. (2018). Enhanced Antioxidant Activity for Apple Juice Fermented with Lactobacillus plantarum ATCC14917. Molecules.

[22] Kiczorowski P., Kiczorowska B., Samolińska W., Szmigielski M., Winiarska-Mieczan A. (2022). Effect of fermentation of chosen vegetables on the nutrient, mineral, and biocomponent profile in human and animal nutrition. Sci. Rep..

[23] Zdziobek P., Jodłowski G.S., Strzelec E.A. (2023). Biopreservation and Bioactivation Juice from Waste Broccoli with Lactiplantibacillus plantarum. Molecules.

[24] Ye J.H., Huang L.Y., Terefe N.S., Augustin M.A. (2019). Fermentation-based biotransformation of glucosinolates, phenolics and sugars in retorted broccoli puree by lactic acid bacteria. Food Chem..

[25] Salehi F., Inanloodoghouz M., Ghazvineh S. (2023). Influence of microwave pretreatment on the total phenolics, antioxidant activity, moisture diffusivity, and rehydration rate of dried sweet cherry. Food Sci. Nutr..

[26] Md Salim N.S., Garièpy Y., Raghavan V. (2019). Effects of Processing on Quality Attributes of Osmo-Dried Broccoli Stalk Slices. Food Bioproc. Technol..

[27] Pandiselvam R., Aydar A.Y., Kutlu N., Aslam R., Sahni P., Mitharwal S., Gavahian M., Kumar M., Raposo A., Yoo S. (2023). Individual and interactive effect of ultrasound pre-treatment on drying kinetics and biochemical qualities of food: A critical review. Ultrason. Sonochem.

[28] Zhu X., Das R.S., Bhavya M.L., Garcia-Vaquero M., Tiwari B.K. (2024). Acoustic cavitation for agri-food applications: Mechanism of action, design of new systems, challenges and strategies for scale-up. Ultrason Sonochem..

[29] Llavata B., García-Pérez J.V., Simal S., Cárcel J.A. (2020). Innovative pre-treatments to enhance food drying: A current review. Curr. Opin. Food Sci..

[30] Salehi F. (2020). Physico-chemical properties of fruit and vegetable juices as affected by ultrasound: A review. Int. J. Food Prop..

[31] Barba F.J., Mariutti L.R.B., Bragagnolo N., Mercadante A.Z., Barbosa-Cánovas G.V., Orlien V. (2017). Bioaccessibility of bioactive compounds from fruits and vegetables after thermal and nonthermal processing. Trends Food Sci. Technol..

[32] Taheri M.E., Salimi E., Saragas K., Novakovic J., Barampouti E.M., Mai S., Malamis D., Moustakas K., Loizidou M. (2021). Effect of pretreatment techniques on enzymatic hydrolysis of food waste. Biomass Convers. Biorefin..

[33] Aamir M., Ovissipour M., Sablani S.S., Rasco B. (2013). Predicting the Quality of Pasteurized Vegetables Using Kinetic Models: A Review. Int. J. Food Sci..

[34] Bas-Bellver C., Barrera C., Betoret N., Seguí L. (2023). Impact of Fermentation Pretreatment on Drying Behaviour and Antioxidant Attributes of Broccoli Waste Powdered Ingredients. Foods.

[35] Liu Y.W., Liong M.T., Tsai Y.C. (2018). New perspectives of *Lactobacillus plantarum* as a probiotic: The gut-heart-brain axis. J. Microbiol..

[36] Seddik H.A., Bendali F., Gancel F., Fliss I., Spano G., Drider D. (2017). Lactobacillus plantarum and Its Probiotic and Food Potentialities. Probiotics Antimicrob. Proteins.

[37] Jiang H., Zhang M., Adhikari B. (2024). Fruits and vegetable powders. Handbook of Food Powders.

[38] Clifford P.A. (1934). Report on Moisture in Dried Fruit. J. AOAC Int..

[39] Singleton V.L., Orthofer R., Lamuela-Raventós R.M. (1999). Analysis of total phenols and other oxidation substrates and antioxidants by means of folin-ciocalteu reagent. Methods Enzym..

[40] Wolfe K., Wu X., Liu R.H. (2003). Antioxidant Activity of Apple Peels. J. Agric. Food Chem..

[41] Luximon-Ramma A., Bahorun T., Soobrattee M.A., Aruoma O.I. (2002). Antioxidant Activities of Phenolic, Proanthocyanidin, and Flavonoid Components in Extracts of *Cassia fistula*. J. Agric. Food Chem..

[42] Brand-Williams W., Cuvelier M.E., Berset C. (1995). Use of a free radical method to evaluate antioxidant activity. LWT Food Sci. Technol..

[43] Re R., Pellegrini N., Proteggente A., Pannala A., Yang M., Rice-Evans C. (1999). Antioxidant activity applying an improved ABTS radical cation decolorization assay. Free Radic. Biol. Med..

[44] Caprioli G., Nzekoue F.K., Giusti F., Vittori S., Sagratini G. (2018). Optimization of an extraction method for the simultaneous quantification of sixteen polyphenols in thirty-one pulse samples by using HPLC-MS/MS dynamic-MRM triple quadrupole. Food Chem..

[45] Giusti F., Capuano E., Sagratini G., Pellegrini N. (2019). A comprehensive investigation of the behaviour of phenolic compounds in legumes during domestic cooking and in vitro digestion. Food Chem..

[46] de Oliveira P.M., de Leite Júnior B.R.C., Martins E.M.F., Martins M.L., Vieira É.N.R., de Barros F.A.R., Cristianini M., de Almeida Costa N., Ramos A.M. (2021). Mango and carrot mixed juice: A new matrix for the vehicle of probiotic lactobacilli. J. Food Sci. Technol..

[47] Septembre-Malaterre A., Remize F., Poucheret P. (2018). Fruits and vegetables, as a source of nutritional compounds and phytochemicals: Changes in bioactive compounds during lactic fermentation. Food Res. Int..

[48] Alp D., Bulantekin Ö. (2021). The microbiological quality of various foods dried by applying different drying methods: A review. Eur. Food Res. Technol..

[49] Lewicki P.P., Pawlak G. (2003). Effect of Drying on Microstructure of Plant Tissue. Dry. Technol..

[50] Djantou E.B., Mbofung C.M.F., Scher J., Phambu N., Morael J.D. (2011). Alternation drying and grinding (ADG) technique: A novel approach for producing ripe mango powder. LWT Food Sci. Technol..

[51] Santos P.H.S., Silva M.A. (2008). Retention of Vitamin C in Drying Processes of Fruits and Vegetables—A Review. Dry. Technol..

[52] Gulati T., Datta A.K. (2015). Mechanistic understanding of case-hardening and texture development during drying of food materials. J. Food Eng..

[53] Kwaw E., Ma Y., Tchabo W., Apaliya M.T., Wu M., Sackey A.S., Xiao L., Tahir H.E. (2018). Effect of lactobacillus strains on phenolic profile, color attributes and antioxidant activities of lactic-acid-fermented mulberry juice. Food Chem..

[54] Yang X., Hong J., Wang L., Cai C., Mo H., Wang J., Fang X., Liao Z. (2024). Effect of Lactic Acid Bacteria Fermentation on Plant-Based Products. Fermentation.

[55] Knez E., Kadac-Czapska K., Grembecka M. (2023). Effect of Fermentation on the Nutritional Quality of the Selected Vegetables and Legumes and Their Health Effects. Life.

[56] Buckenhueskes H.J. (2015). Quality improvement and fermentation control in vegetables. Advances in Fermented Foods and Beverages: Improving Quality, Technologies and Health Benefits.

[57] Chin S., Siew E., Soon W. (2015). Drying characteristics and quality evaluation of kiwi slices under hot air natural convective drying method. Int. Food Res. J..

[58] Chen M.L., Yang D.J., Liu S.C. (2011). Effects of drying temperature on the flavonoid, phenolic acid and antioxidative capacities of the methanol extract of citrus fruit (*Citrus sinensis* (L.) Osbeck) peels. Int. J. Food Sci. Technol..

[59] Bernaert N., De Clercq H., Van Bockstaele E., De Loose M., Van Droogenbroeck B. (2013). Antioxidant changes during postharvest processing and storage of leek (*Allium ampeloprasum* var. porrum). Postharvest Biol. Technol..

[60] Papoutsis K., Pristijono P., Golding J.B., Stathopoulos C.E., Bowyer M.C., Scarlett C.J., Vuong Q.V. (2017). Effect of vacuum-drying, hot air-drying and freeze-drying on polyphenols and antioxidant capacity of lemon (*Citrus limon*) pomace aqueous extracts. Int. J. Food Sci. Technol..

[61] Lutz M., Hernández J., Henríquez C. (2015). Phenolic content and antioxidant capacity in fresh and dry fruits and vegetables grown in Chile. CYTA J. Food.

[62] Nooshkam M., Varidi M., Bashash M. (2019). The Maillard reaction products as food-born antioxidant and antibrowning agents in model and real food systems. Food Chem..

[63] Honda M., Kageyama H., Hibino T., Ichihashi K., Takada W., Goto M. (2020). Isomerization of Commercially Important Carotenoids (Lycopene, β-Carotene, and Astaxanthin) by Natural Catalysts: Isothiocyanates and Polysulfides. J. Agric. Food Chem..

[64] Chamberlain M.C., O’Flaherty S., Cobián N., Barrangou R. (2022). Metabolomic Analysis of *Lactobacillus acidophilus*, L. *gasseri*, L. *crispatus*, and *Lacticaseibacillus rhamnosus* Strains in the Presence of Pomegranate Extract. Front. Microbiol..

[65] Marques L.G., Silveira A.M., Freire J.T. (2006). Freeze-Drying Characteristics of Tropical Fruits. Dry. Technol..

[66] Silva-Espinoza M.A., Ayed C., Foster T., Del Mar Camacho M., Martínez-Navarrete N. (2019). The Impact of Freeze-Drying Conditions on the Physico-Chemical Properties and Bioactive Compounds of a Freeze-Dried Orange Puree. Foods.

[67] Tylewicz U., Nowacka M., Rybak K., Drozdzal K., Dalla Rosa M., Mozzon M. (2020). Design of Healthy Snack Based on Kiwifruit. Molecules.

[68] Dalmau M.E., Bornhorst G.M., Eim V., Rosselló C., Simal S. (2017). Effects of freezing, freeze drying and convective drying on in vitro gastric digestion of apples. Food Chem..

[69] Rudy S., Dziki D., Krzykowski A., Gawlik-Dziki U., Polak R., Rózyło R., Kulig R. (2015). Influence of pre-treatments and freeze-drying temperature on the process kinetics and selected physico-chemical properties of cranberries (*Vaccinium macrocarpon* Ait.). LWT Food Sci. Technol..

[70] Bas-Bellver C., Barrera C., Betoret N., Seguí L. (2023). Effect of Processing and In Vitro Digestion on Bioactive Constituents of Powdered IV Range Carrot (*Daucus carota*, L.). Wastes. Foods.

[71] Cui L., Niu L., Li D., Liu C., Liu Y., Liu C., Song J. (2018). Effects of different drying methods on quality, bacterial viability and storage stability of probiotic enriched apple snacks. J. Integr. Agric..

[72] Champagne C.P., Ross R.P., Saarela M., Hansen K.F., Charalampopoulos D. (2011). Recommendations for the viability assessment of probiotics as concentrated cultures and in food matrices. Int. J. Food Microbiol..

[73] Betoret E., Betoret N., Arilla A., Bennár M., Barrera C., Codoñer P., Fito P. (2012). No invasive methodology to produce a probiotic low humid apple snack with potential effect against Helicobacter pylori. J. Food Eng..

[74] Shekh S.L., Boricha A.A., Chavda J.G., Vyas B.R.M. (2020). Probiotic potential of lyophilized *Lactobacillus plantarum*, G.P. Ann Microbiol..

[75] Rishabh D., Athira A., Preetha R., Nagamaniammai G. (2023). Freeze dried probiotic carrot juice powder for better storage stability of probiotic. J. Food Sci. Technol..

[76] Bekić Šarić B. Processing of agricultural products by lyophilization. Proceedings of the II International Scientific Conference “Sustainable Agriculture and Rural Development”.

[77] Guiné R.P.F. (2018). The Drying of Foods and Its Effect on the Physical-Chemical, Sensorial and Nutritional Properties. ETP Int. J. Food Eng..

[78] Scherzinger M., Kulbeik T., Kaltschmitt M. (2020). Autoclave pre-treatment of green wastes—Effects of temperature, residence time and rotation speed on fuel properties. Fuel.

[79] Guo Q., Sun D.W., Cheng J.H., Han Z. (2017). Microwave processing techniques and their recent applications in the food industry. Trends Food Sci. Technol..

[80] Ye M., Zhou H., Hao J., Chen T., He Z., Wu F., Liu X. (2021). Microwave pretreatment on microstructure, characteristic compounds and oxidative stability of Camellia seeds. Ind. Crops Prod..

[81] Kong F., Singh R.P. (2016). Chemical Deterioration and Physical Instability of Foods and Beverages. The Stability and Shelf Life of Food.

[82] Rolfe C., Daryaei H. (2020). Intrinsic and Extrinsic Factors Affecting Microbial Growth in Food Systems. Food Safety Engineering.

[83] Nimkarde A.D., Gopnarayan S.P., Vaidya K.S. (2022). Effect of Microwaves on the pH and °Brix value of Cranberry, Grape, Blackberry and Lemon. J. Adv. Appl. Sci. Res..

[84] Alvi T., Khan M.K.I., Maan A.A., Shahid M., Sablani S. (2023). Microwaves as sustainable approach for artificial ripening of date fruit cv. Khupra Reduce Fruit. Waste. Food Biosci..

[85] Malik F., Nadeem M., Ainee A., Kanwal R., Sultan M., Iqbal A., Mahmoud S.F., Alshehry G.A., Jumayi H.A.A.L., Algarni E.H.A. (2022). Quality Evaluation of Lemon Cordial Stored at Different Times with Microwave Heating (Pasteurization). Sustainability.

[86] Conesa C., Seguí L., Laguarda-Miró N., Fito P. (2016). Microwaves as a pretreatment for enhancing enzymatic hydrolysis of pineapple industrial waste for bioethanol production. Food Bioprod. Process..

[87] Shrotri A., Kobayashi H., Fukuoka A. (2018). Cellulose Depolymerization over Heterogeneous Catalysts. Acc. Chem. Res..

[88] Liu H., Ni Y., Yu Q., Fan L. (2023). Evaluation of co-fermentation of L. plantarum and P. kluyveri of a plant-based fermented beverage: Physicochemical, functional, and sensory properties. Food Res. Int..

[89] Peng W., Meng D., Yue T., Wang Z., Gao Z. (2021). Effect of the apple cultivar on cloudy apple juice fermented by a mixture of Lactobacillus acidophilus, Lactobacillus plantarum, and *Lactobacillus fermentum*. Food Chem..

[90] Santhirasegaram V., Razali Z., Somasundram C. (2013). Effects of thermal treatment and sonication on quality attributes of Chokanan mango (*Mangifera indica* L.) juice. Ultrason. Sonochem.

[91] Aadil R.M., Zeng X.A., Wang M.S., Liu Z.W., Han Z., Zhang Z.H., Hong J., Jabbar S. (2015). A potential of ultrasound on minerals, micro-organisms, phenolic compounds and colouring pigments of grapefruit juice. Int. J. Food Sci. Technol..

[92] Wang H., Zhao Q.S., Wang X.D., Hong Z dong Zhao B. (2019). Pretreatment of ultrasound combined vacuum enhances the convective drying efficiency and physicochemical properties of okra (*Abelmoschus esculentus*). LWT.

[93] Santos N.C., Almeida R.L.J., Albuquerque J.C., de Andrade E.W.V., Gregório M.G., Santos R.M.S., Rodrigues T.J.A., Carvalho R.d.O., Gomes M.M.d.A., Moura H.V. (2024). Optimization of ultrasound pre-treatment and the effect of different drying techniques on antioxidant capacity, bioaccessibility, structural and thermal properties of purple cabbage. Chem. Eng. Process. Process Intensif..

[94] Urquieta-Herrero M., Cornejo-Mazón M., Gutiérrez-López G.F., García-Pinilla S. (2021). Effect of two pasteurization methods on the content of bioactive compounds and antioxidant capacity of nance (*Byrsonima crassifolia*) pulp and their kinetics of loss during refrigerated storage. Rev. Mex. Ing. Quim..

[95] Alongi M. (2020). Sviluppo di Alimenti Funzionali Mediante Interventi di Proceso e Formulazione Innovativi e Sostenibili.

[96] Debelo H., Li M., Ferruzzi M.G. (2020). Processing influences on food polyphenol profiles and biological activity. Curr. Opin. Food Sci..

[97] Cai Y.X., Wang J.H., McAuley C., Augustin M.A., Terefe N.S. (2019). Fermentation for enhancing the bioconversion of glucoraphanin into sulforaphane and improve the functional attributes of broccoli puree. J. Funct. Foods.

[98] Meng F.B., Lei Y.T., Li Q.Z., Li Y.C., Deng Y., Liu D.Y. (2022). Effect of *Lactobacillus plantarum* and *Lactobacillus acidophilus* fermentation on antioxidant activity and metabolomic profiles of loquat juice. LWT.

[99] Garcia C., Remize F. (2022). Lactic acid fermentation of fruit and vegetable juices and smoothies: Innovation and health aspects. Lactic Acid Bacteria in Food Biotechnology—Innovations and Functional Aspects.

[100] Sharma R., Garg P., Kumar P., Bhatia S.K., Kulshrestha S. (2020). Microbial Fermentation and Its Role in Quality Improvement of Fermented Foods. Fermentation.

[101] Hou F., Cai Y., Wang J. (2023). Antioxidant Capacity Changes and Untargeted Metabolite Profile of Broccoli during Lactic Acid Bacteria Fermentation. Fermentation.

[102] Bei Q., Liu Y., Wang L., Chen G., Wu Z. (2017). Improving free, conjugated, and bound phenolic fractions in fermented oats (*Avena sativa* L.) with Monascus anka and their antioxidant activity. J. Funct. Foods.

[103] Wu C., Li T., Qi J., Jiang T., Xu H., Lei H. (2020). Effects of lactic acid fermentation-based biotransformation on phenolic profiles, antioxidant capacity and flavor volatiles of apple juice. LWT.

[104] Sapci Z. (2013). The effect of microwave pretreatment on biogas production from agricultural straws. Bioresour. Technol..

[105] Almaiman S.A., Albadr N.A., Alsulaim S., Alhuthayli H.F., Osman M.A., Hassan A.B. (2021). Effects of microwave heat treatment on fungal growth, functional properties, total phenolic content, and antioxidant activity of sorghum (*Sorghum bicolor* L.) grain. Food Chem..

[106] Álvarez A., Poejo J., Matias A.A., Duarte C.M.M., Cocero M.J., Mato R.B. (2017). Microwave pretreatment to improve extraction efficiency and polyphenol extract richness from grape pomace. Effect on antioxidant bioactivity. Food Bioprod. Process..

[107] Gupta Y., Barrett B., Vlachos D.G. (2024). Understanding microwave-assisted extraction of phenolic compounds from diverse food waste feedstocks. Chem. Eng. Process. Process Intensif..

[108] Popoola O.O. (2022). Phenolic compounds composition and in vitro antioxidant activity of Nigerian Amaranthus viridis seed as affected by autoclaving and germination. Meas. Food.

[109] Sanchiz A., Pedrosa M.M., Guillamón E., Arribas C., Cabellos B., Linacero R., Cuadrado C. (2019). Influence of boiling and autoclave processing on the phenolic content, antioxidant activity and functional properties of pistachio, cashew and chestnut flours. LWT.

[110] Rojas M.L., Kubo M.T.K., Caetano-Silva M.E., Augusto P.E.D. (2021). Ultrasound processing of fruits and vegetables, structural modification and impact on nutrient and bioactive compounds: A review. Int. J. Food Sci. Technol..

[111] Kumar K., Srivastav S., Sharanagat V.S. (2021). Ultrasound assisted extraction (UAE) of bioactive compounds from fruit and vegetable processing by-products: A review. Ultrason. Sonochem.

[112] Huang D., Men K., Li D., Wen T., Gong Z., Sunden B., Wu Z. (2020). Application of ultrasound technology in the drying of food products. Ultrason. Sonochem.

[113] Rajewska K., Mierzwa D. (2017). Influence of ultrasound on the microstructure of plant tissue. Innov. Food Sci. Emerg. Technol..

[114] Vera Zambrano M., Dutta B., Mercer D.G., MacLean H.L., Touchie M.F. (2019). Assessment of moisture content measurement methods of dried food products in small-scale operations in developing countries: A review. Trends Food Sci. Technol..

[115] Prosapio V., Norton I. (2017). Influence of osmotic dehydration pre-treatment on oven drying and freeze drying performance. LWT.

[116] Maskan M. (2001). Drying, shrinkage and rehydration characteristics of kiwifruits during hot air and microwave drying. J. Food Eng..

[117] Janiszewska-Turak E., Rybak K., Pobiega K., Nikodem A., Gramza-Michałowska A. (2022). Sustainable Production and Characteristics of Dried Fermented Vegetables. Fermentation.

[118] Oyinloye T.M., Yoon W.B. (2020). Effect of Freeze-Drying on Quality and Grinding Process of Food Produce: A Review. Processes.

[119] Ramírez-Pulido B., Bas-Bellver C., Betoret N., Barrera C., Seguí L. (2021). Valorization of Vegetable Fresh-Processing Residues as Functional Powdered Ingredients. A Review on the Potential Impact of Pretreatments and Drying Methods on Bioactive Compounds and Their Bioaccessibility. Front. Sustain. Food Syst..

[120] Huang Y., Sun Y., Mehmood A., Lu T., Chen X. (2024). Unraveling the temporal changes of Maillard reaction products and aroma profile in coffee leaves during hot-air drying. J. Food Compos. Anal..

[121] Somjai C., Siriwoharn T., Kulprachakarn K., Chaipoot S., Phongphisutthinant R., Wiriyacharee P. (2021). Utilization of Maillard reaction in moist-dry-heating system to enhance physicochemical and antioxidative properties of dried whole longan fruit. Heliyon.

[122] Réblová Z. (2012). Effect of temperature on the antioxidant activity of phenolic acids. Czech J. Food Sci..

[123] Parchem K., Piekarska A., Bartoszek A. (2020). Enzymatic activities behind degradation of glucosinolates. Glucosinolates: Properties, Recovery, and Applications.

[124] Antal T. (2015). Comparative study of three drying methods: Freeze, hot air-assisted freeze and infrared-assisted freeze modes. Agron. Res..

[125] Vargas L., Kapoor R., Nemzer B., Feng H. (2022). Application of different drying methods for evaluation of phytochemical content and physical properties of broccoli, kale, and spinach. LWT.

[126] Zura-Bravo L., Rodriguez A., Stucken K., Vega-Gálvez A. (2019). Drying kinetics of probiotic-impregnated murta (*Ugni molinae* T.) berries. J. Food Sci. Technol..

[127] Lee Y., Oh J., Jeong Y.S. (2015). Lactobacillus plantarum-mediated conversion of flavonoid glycosides into flavonols, quercetin, and kaempferol in Cudrania tricuspidata leaves. Food Sci. Biotechnol..

[128] Filannino P., Bai Y., Di Cagno R., Gobbetti M., Gänzle M.G. (2015). Metabolism of phenolic compounds by Lactobacillus spp. during fermentation of cherry juice and broccoli puree. Food Microbiol..

[129] Vega-Galvez A., Uribe E., Pasten A., Camus J., Rojas M., Garcia V., Araya M., Valenzuela-Barra G., Zambrano A., Goñi M.G. (2023). Low-Temperature Vacuum Drying on Broccoli: Enhanced Anti-Inflammatory and Anti-Proliferative Properties Regarding Other Drying Methods. Foods.

[130] Chu Q., Li L., Duan X., Zhao M., Wang Z., Wang Z., Ren X., Li C., Ren G. (2023). Effect mechanism of different drying methods on the quality and browning for daylily. LWT.

